# Nuclear and Cytoplasmic Accumulation of Ep-ICD Is Frequently Detected in Human Epithelial Cancers

**DOI:** 10.1371/journal.pone.0014130

**Published:** 2010-11-30

**Authors:** Ranju Ralhan, Helen C.-H. He, Anthony K.-C. So, Satyendra C. Tripathi, Manish Kumar, Md. Raghibul Hasan, Jatinder Kaur, Lawrence Kashat, Christina MacMillan, Shyam Singh Chauhan, Jeremy L. Freeman, Paul G. Walfish

**Affiliations:** 1 Joseph and Mildred Sonshine Family Centre for Head and Neck Diseases, Department of Otolaryngology-Head and Neck Surgery Program, Mount Sinai Hospital, Toronto, Ontario, Canada; 2 Alex and Simona Shnaider Laboratory in Molecular Oncology, Department of Pathology and Laboratory Medicine, Mount Sinai Hospital, Joseph and Wolf Lebovic Health Complex, Toronto, Ontario, Canada; 3 Department of Pathology and Laboratory Medicine, Mount Sinai Hospital, Joseph and Wolf Lebovic Health Complex, Toronto, Ontario, Canada; 4 Endocrine Division, Department of Medicine, Mount Sinai Hospital and University of Toronto Medical School, Toronto, Ontario, Canada; 5 Department of Otolaryngology-Head and Neck Surgery, University of Toronto, Toronto, Ontario, Canada; 6 Department of Biochemistry, All India Institute of Medical Sciences, New Delhi, India; 7 Institute of Medical Science, University of Toronto, Toronto, Ontario, Canada; University Medical Center Maastricht, Netherlands

## Abstract

**Background:**

We previously demonstrated that nuclear and cytoplasmic accumulation of the intracellular domain (Ep-ICD) of epithelial cell adhesion molecule (EpCAM) accompanied by a reciprocal reduction of its extracellular domain (EpEx), occurs in aggressive thyroid cancers. This study was designed to determine whether similar accumulation of Ep-ICD is a common event in other epithelial cancers.

**Methodology and Results:**

Ten epithelial cancers were immunohistochemically analyzed using Ep-ICD and EpEx domain-specific antibodies. The subcellular localization of EpEx and Ep-ICD in the human colon adenocarcinoma cell line CX-1 was observed using immunofluorescence. Nuclear and cytoplasmic Ep-ICD expression was increased in cancers of the breast (31 of 38 tissues, 82%), prostate (40 of 49 tissues, 82%), head and neck (37 of 57 tissues, 65%) and esophagus (17 of 46 tissues, 37%) compared to their corresponding normal tissues that showed membrane localization of the protein. Importantly, Ep-ICD was not detected in the nuclei of epithelial cells in most normal tissues. High nuclear and cytoplasmic Ep-ICD accumulation also occurred in the other six epithelial cancer types analyzed - lung, colon, liver, bladder, pancreatic, and ovarian. A concomitant reduction in membrane EpEx expression was observed in a subset of all cancer types. Receiver operating characteristic curve analysis revealed nuclear Ep-ICD distinguished breast cancers with 82% sensitivity and 100% specificity and prostate cancers with 82% sensitivity and 78% specificity. Similar findings were observed for cytoplasmic accumulation of Ep-ICD in these cancers. We provide clinical evidence of increased nuclear and cytoplasmic Ep-ICD accumulation and a reduction in membranous EpEx in these cancers.

**Conclusions:**

Increased nuclear and cytoplasmic Ep-ICD was observed in all epithelial cancers analyzed and distinguished them from normal tissues with high-sensitivity, specificity, and AUC. Development of a robust high throughput assay for Ep-ICD will facilitate the determination of its diagnostic, prognostic and therapeutic relevance in epithelial cancers.

## Introduction

Epithelial cell adhesion molecule (EpCAM) is a 40 kDa transmembrane glycoprotein that serves important roles in cell adhesion, cell proliferation, differentiation, migration, cell cycle regulation and is implicated in cancer and stem cell signalling [1]. EpCAM is one of the most widely investigated proteins in human cancers, frequently overexpressed in human malignancies, localized on the plasma membrane of tumor cells and albeit at lower levels in the normal epithelia [2], [3], [4], [5], [6], [7], [8], [9], [10], [11], [12], [13], [14], [15], [16], [17]. All these studies used antibodies directed against the extracellular domain of EpCAM (EpEx) [13]. These numerous reports on the cell surface expression of EpCAM in human cancers have suggested that it could be an ideal candidate for application as an epithelial cancer marker and a therapeutic target [18], [19], [20], [21]. Paradoxically, most clinical trials using murine monoclonal antibodies namely, edrecolomab in colorectal cancer, or the humanized antibody, adecatumumab, in breast cancer have shown limited efficacy [14], [22]. An understanding of these limitations poses a challenge for oncologists and is of great importance for future development of more effective anti-EpCAM strategies. In this context, Gires and Baeuerle [23] discussed the need to measure EpCAM expression levels in tumor cells and their impact on the outcome of a clinical trial. However, none of the previous trials have analyzed EpCAM expression in tumor tissues, prospectively or retrospectively. Whether the recently reported regulated intramembrane proteolysis (RIP) mediated loss of EpCAM from the tumor cell surface might be one of the reasons for the limited efficacy of EpCAM-based cancer therapies remains to be established [24]. The cleavage of the EpCAM ectodomain, EpEx, by the protease tumor necrosis factor α converting enzyme (TACE) and its shedding has been shown to release its intracellular domain (Ep-ICD) which then translocates to the nucleus resulting in activation of oncogenic signalling [24]. The association of Ep-ICD with FHL2 and Wnt pathway components β-catenin and Lef-1 forms a nuclear complex that binds DNA at Lef-1 consensus sites and induces gene transcription, leading to increased cell proliferation [24]. The clinical significance of Ep-ICD in human cancers needs to be determined in view of the multiple roles of EpCAM as an oncogenic signal transducer, cell adhesion molecule and cancer stem cell marker [24], [25], [26], [27].

Nuclear localization of Ep-ICD was first reported in a study of 26 cases of human colon cancer, but not in normal colonic epithelia [24]. Subsequently, we reported nuclear and cytoplasmic accumulation of Ep-ICD in different subtypes of thyroid cancers that was associated with a reciprocal reduction in membranous EpEx, and also observed a correlation of nuclear Ep-ICD accumulation with tumor aggressiveness and poor disease prognosis [28]. The wide heterogeneity in solid tumors warrants investigation to determine whether nuclear and cytoplasmic Ep-ICD expression may also occur in other human cancers. In the current study, the subcellular compartmental accumulation of Ep-ICD has been addressed in a wide variety of epithelial cancers, namely, breast, prostate, head and neck, esophagus, ovary, pancreas, colon and rectum, lung, urinary bladder, liver carcinomas by immunohistochemistry (IHC) (using a specific antibody directed against the Ep-ICD domain of EpCAM). Nuclear and cytoplasmic Ep-ICD has also been quantitatively detected in CX-1 colon cancer cells using immunofluorescence. With the exception of a previous report in colon cancer and our study in thyroid cancer [24], [28], the novelty of this report is the demonstration of the widespread occurrence of increased nuclear and cytoplasmic accumulation of Ep-ICD in association with variable membrane EpEx expression in a wide spectrum of epithelial cancers.

## Methods

### Ethics Statement

This study was conducted according to the principles expressed in the Declaration of Helsinki. The study was approved by Mount Sinai Hospital Research Ethics Board, Toronto, Canada. The archived paraffin tissue blocks of a head and neck cancer study approved by the Human Ethics Committee of All India Institute of Medical Sciences, New Delhi, India, with prior consent of the patients, were used in this study.

### Patients and tissue specimens

For IHC analysis, archived tissue blocks of head and neck squamous cell carcinomas (HNSCCs) and normal tissues as well as esophageal squamous cell carcinomas (ESCCs) were retrieved from the tumor bank, reviewed by the pathologist and used for cutting tissue sections for immunostaining with Ep-ICD and EpEx antibodies as described below. The clinical and pathological data recorded included clinical tumor stage, site of the lesions, histopathological differentiation, age and gender in a pre-designed performa as described by us previously [29]. Tissue-microarrays (TMA) of breast cancer and adjacent normal breast tissue (IMH-371), prostate cancer and adjacent normal prostate tissue (IMH-303), lung cancer (IMT-305), colon and rectal cancer (IMH-306), common epithelial cancers comprising of liver, urinary bladder, ovaries, pancreas, breast and prostate (IMH-327) were procured from Imgenex Corp (San Diego, CA). Twenty-one tissue blocks of benign prostate hyperplasia (BPH) were retrieved from the tissue bank in Mount Sinai Hospital, reviewed by the pathologist and used for cutting tissue sections for immunostaining with Ep-ICD and EpEx antibodies.

### Antibodies and Cell Line

Anti-human Ep-ICD rabbit monoclonal antibody was obtained from Epitomics Inc. (Burlingame, CA). Anti-human EpEx mouse monoclonal antibody (MOC-31) was obtained from AbD serotec (Raleigh, NC). The α-Ep-ICD antibody 1144 recognizes the cytoplasmic domain of human EpCAM and has been used in our recent study on Ep-ICD in thyroid cancer [28]. MOC-31 recognizes the extracellular domain of EpCAM. Both antibodies have been used in our recent study in thyroid cancer [28].

The human colon adenocarcinoma cell line CX-1 was cultured in RPMI-1640 media containing 100 µg/mL streptomycin and 100 U/mL penicillin, 10% fetal bovine serum (FBS) and 1% non-essential amino acids. The STR profile of the cell line was found to be in accordance with the known profile of CX-1 in the databases of the German Collection of Microorganisms and Cell Cultures (DSMZ, Braunschweig, Germany).

### Clinicopathological characteristics of patients

Fifty-seven HNSCC patients, ranging in age from 29 to 75 years were enrolled in this study. Their diagnoses were based on clinical examination and histopathological analysis of the tissue specimens. The tumors were histologically graded as well differentiated squamous cell carcinoma (WDSCC), moderately differentiated squamous cell carcinoma (MDSCC) or poorly differentiated squamous cell carcinoma (PDSCC). Twenty tissues taken from a distant site of HNSCCs (with histologically confirmed normal epithelia referred to here as head and neck normal tissues) were also evaluated for Ep-ICD and EpEx expression.

Forty-six ESCC patients were enrolled in this study. Their diagnoses were based on clinical examination and histopathological analysis of the tissue specimens. The tumors were histologically graded as well, moderately or poorly differentiated SCCs. Twenty tissues taken from a distant site of ESCCs (with histologically confirmed normal epithelia referred to here as esophageal normal tissues) were also evaluated for Ep-ICD protein expression. Similarly, forty tissues taken from a distant site of ESCCs (with histologically confirmed normal epithelia referred to here as esophageal normal tissues) were evaluated for EpEx expression.

TMAs of breast cancer and adjacent normal breast tissue, prostate cancer and adjacent normal prostate tissue, lung cancer, colon and rectal cancer, common epithelial cancers comprising of liver, urinary bladder, ovaries, pancreas, breast and prostate were examined. For Ep-ICD, the number of tissues analyzed were 38 breast cancers and 25 corresponding normal tissues, 49 prostate cancers and 9 corresponding normal tissues and 21 benign prostate hyperplasias, 57 HNSCCs and 20 corresponding normal tissues, 46 ESCCs and 20 corresponding normal tissues, 59 each of lung and colon cancers, 10 each of bladder, ovarian and pancreatic cancers, and 9 liver cancers. For EpEx, the number of tissues that were available for immunohistochemical analysis included 40 breast cancers and 29 corresponding normal tissues, 49 prostate cancers and 9 corresponding normal tissues and 21 benign prostate hyperplasias, 39 HNSCCs and 20 corresponding normal tissues, 47 ESCCs and 40 corresponding normal tissues, 59 cases each of lung and colon cancers, and 10 cases each of bladder, ovarian, and liver cancers.

### Immunohistochemical analysis of Ep-ICD expression in epithelial cancers

Serial paraffin-embedded sections (5 µm thickness) of HNSCCs, ESCCs and their normal tissues were used for Ep-ICD and EpEx immunostaining as we have described recently [28]. The TMA slides and the individual tissue sections were de-paraffinized by baking at 62°C for 1 hour in vertical orientation and rehydrated in xylene and graded alcohol series. Thereafter, antigen retrieval conditions were optimized and was carried out in 0.01 M citrate buffer, pH 6.0 for 3 minutes at 115°C, using a TTmega oven (Milestone Inc. Shelton, CT). Endogenous peroxidase activity was blocked by incubating sections in methanol containing 0.3% hydrogen peroxide for 20 minutes. After blocking the non-specific binding with normal horse or goat serum, the sections were incubated with α- Ep-ICD rabbit monoclonal antibody 1144 (dilution 1∶200) for 30 minutes and biotinylated goat anti-rabbit secondary antibody for 30 minutes or incubated with MOC-31 (dilution 1∶200) for 30 minutes with the corresponding biotinylated secondary antibody (horse anti-mouse or goat anti-rabbit) (Vector Laboratories, Burlington, Ontario, Canada) for 30 minutes. The sections were finally incubated with VECTASTAIN Elite ABC Reagent (Vector Laboratories) and diaminobenzedine was used as the chromogen.

### Evaluation of immunohistochemical staining

The TMA images were acquired using the Visiopharm Integrator System (Visiopharm, Horsholm, Denmark). Ep-ICD and EpEx immunopositive staining was evaluated in five areas of the acquired images of the tissue sections and the average of these five scores was calculated. Sections were scored as positive if epithelial cells showed immunopositivity in the plasma membrane, cytoplasm, and/or nucleus when observed by two evaluators who were blinded to the clinical histopathology and diagnosis. These sections were scored on the basis of percentage positivity as follows: 0, <10% cells; 1, 10–30% cells; 2, 30–50% cells; 3, 50–70% cells; and 4, >70% cells showed immunoreactivity as described earlier [28]. Sections were also scored semi-quantitatively on the basis of intensity as follows: 0, none; 1, mild; 2, moderate; and 3, intense. Finally, a total score (ranging from 0 to 7) was obtained by adding the scores of percentage positivity and intensity for each cancer and normal tissue section. The immunohistochemical data were subjected to statistical analysis as described previously [29].

### Statistical analysis

The IHC data were subjected to statistical analysis using SPSS 17.0 software (SPSS, Chicago, IL) and Graphpad Prism 5 (GraphPad Software, La Jolla, CA). Scatter plots were used to determine the distribution of total score for membrane, nuclear, and cytoplasmic Ep-ICD expression in normal and cancerous tissues. Receiver operating characteristic (ROC) curve analyses were performed to calculate the sensitivity, specificity and area under the curve (AUC) values in each cancer type for nuclear and cytoplasmic Ep-ICD. Based on the optimal sensitivity and specificity, an IHC score cut-off value of ≥4 was defined as immunopositive uniformly for all the cancer types analyzed for statistical examination.

### Immunofluorescence analysis of Ep-ICD and EpEx localization in CX-1 colon cancer cell line

CX-1 colon cancer cells were grown on glass slides up to 60% confluence and then incubated with either α-Ep-ICD rabbit monoclonal antibody 1144 (1∶100 dilution) or mouse monoclonal antibody MOC-31 (1∶100). For Ep-ICD, the secondary antibody used was a tetramethyl rhodamine isothiocyanate (TRITC)-labeled goat anti-rabbit antibody (Sigma-Aldrich, 1∶200 dilution). For EpEx, the secondary antibody used was a fluorescein isothiocyanate (FITC)-labeled goat anti-mouse antibody (Sigma-Aldrich, St. Louis, MO, 1∶200 dilution). Slides were viewed using an Olympus Upright fluorescence microscope (BX61) and images were analyzed using Volocity software (PerkinElmer, Waltham, MA).

## Results

### Immunohistochemical analysis of Ep-ICD expression in human epithelial cancers

The known clinical parameters, histopathology, and Ep-ICD IHC scores for each cancer type are provided in the Supplementary Tables S1, S2, S3, S4, S5, S6, S7, S8, S9, S10. Among the cancer types that were compared to the normal tissues (Table 1), breast cancers exhibited 82% nuclear Ep-ICD positivity (31 of 38 tissues) and 84% cytoplasmic Ep-ICD positivity (32 of 38 tissues). Based on an IHC cut-off score of 4, none of the 25 normal breast tissues analyzed were considered positive for nuclear Ep-ICD; 56% (14 of 25 tissues) showed no detectable nuclear staining in breast lobular cells or ductal cells, while the remaining 11 cases showed low IHC scores. Prostate cancers exhibited 82% nuclear positivity (40 of 49 tissues) and 82% cytoplasmic positivity (40 of 49 tissues). Nuclear Ep-ICD was positive in 2 of 9 normal prostate tissues, and cytoplasmic positivity was observed in 1 of 9 tissues. Among the 21 BPH tissues analyzed, cytoplasmic positivity was observed in 1 of 21 tissues; none of the tissues were nuclear Ep-ICD positive based on a cutoff score of 4. In HNSCCs, Ep-ICD nuclear positivity was 65% (37 of 57 tissues) and cytoplasmic positivity was 74% (42 of 57 tissues). ESCC nuclear positivity was 37% (17 of 46 tissues) and cytoplasmic positivity was 70% (32 of 37 tissues). Nuclear and cytoplasmic Ep-ICD was each observed to be positive in only 1 of 20 normal tissues in head and neck cancer. For ESCC, nuclear positivity was observed in 2 of 20 normal tissues and cytoplasmic positivity was observed in 6 of 20 normal tissues. All remaining epithelial cancers displayed nuclear and cytoplasmic accumulation of Ep-ICD (Table 1). Notably, although the use of a cutoff of ≥4 to determine positivity led to reduced nuclear and cytoplasmic positivity in pancreatic cancers, an examination of the scoring revealed that a cutoff of 3.5 would have identified 7 of 10 cases of nuclear Ep-ICD accumulation and 5 of 10 cases of cytoplasmic Ep-ICD accumulation (See Supplementary Table S8).

**Table 1:** Summary of Immunohistochemical Analysis of Ep-ICD in Normal and Epithelial Cancers.

Tissue Type	Cancer or Normal	Number Tissues (n)	Nuclear Positive (n)	Nuclear Positivity (%)	Cytoplasmic Positive (n)	Cytoplasmic Positivity (%)
**Prostate**	**Cancer**	49	40	82	40	82
	**Normal**	9	2	22	1	11
	**BPH**	21	0	0	1	5
**Breast**	**Cancer**	38	31	82	32	84
	**Normal**	25	0	0	0	0
**Head and Neck**	**Cancer**	57	37	65	42	74
	**Normal**	20	1	5	1	5
**Esophagus**	**Cancer**	46	17	37	32	70
	**Normal**	20	2	10	6	30
**Lung**	**Cancer**	59	47	80	56	95
**Colon**	**Cancer**	59	49	83	46	78
**Ovary**	**Cancer**	10	10	100	10	100
**Pancreas**	**Cancer**	10	3	30	2	20
**Liver**	**Cancer**	9	9	100	8	89
**Bladder**	**Cancer**	10	9	90	9	90

**Figure 1:**
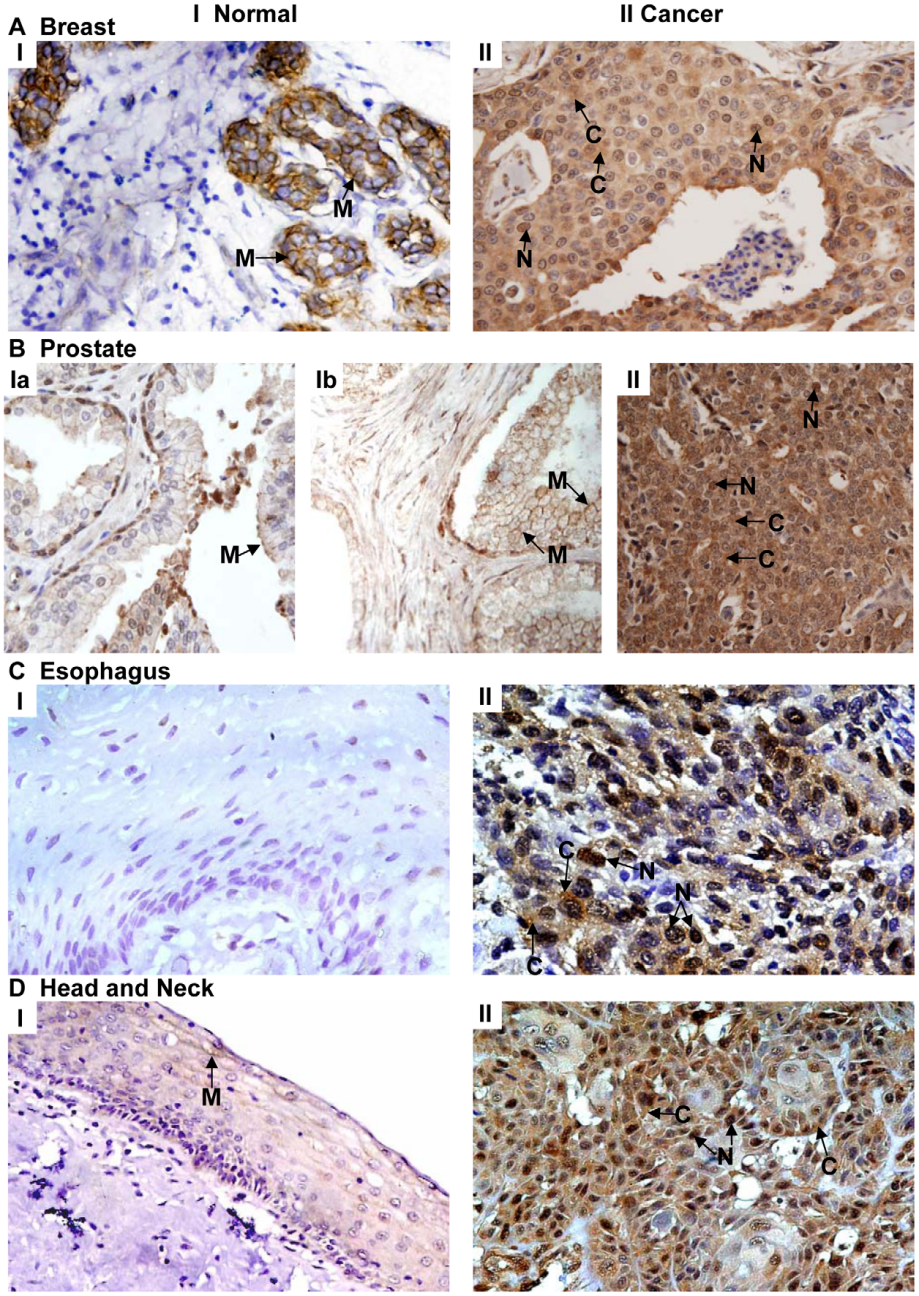
Immunohistochemical analysis of Ep-ICD expression in epithelial cancers and normal tissues. The representative photomicrographs depict Ep-ICD immunostaining in normal and cancer tissues. Panel A shows predominant membrane localization of Ep-ICD and no nuclear staining in the normal breast tissue (I), while the cancer tissue shows nuclear and cytoplasmic Ep-ICD accumulation (II). Panel B shows low level of membrane Ep-ICD in the epithelial cells and the basal cells show some nuclear staining in the normal prostate tissue (Ia) and in benign prostate hyperplasia (B, Ib), while the cancer tissue shows intense cytoplasmic and nuclear staining (II). Panel C shows no detectable Ep-ICD staining in the normal esophageal tissue (I), while the ESCC shows intense nuclear and cytoplasmic immunostaining (II). Panel D depicts head and neck normal mucosa showing faint membrane Ep-ICD (I), while the HNSCC shows intense nuclear and cytoplasmic immunostaining (II). Original magnification ×400.

The representative photomicrographs shown in Figure 1 demonstrate Ep-ICD immunostaining in normal and cancer tissues. Panel A shows predominant membrane localization of Ep-ICD and no nuclear staining in the normal breast tissue (I), while the cancer tissue shows nuclear and cytoplasmic Ep-ICD accumulation (II). Panel B (Ia) depicts low level of membrane Ep-ICD in the epithelial cells and the basal cells show slight nuclear staining in the normal prostate tissue and in benign prostate hyperplasia (Ib). The prostate cancer tissue shows intense cytoplasmic and increased nuclear staining (panel B, II). In endothelial cells, strong Ep-ICD staining was consistently observed. In adipocytes, staining ranged from absent to mild in some cells. Lymphocytes stained strongly in tumor tissues, whereas none was observed in normal tissues. Smooth muscle staining was absent or weak in all cases. No detectable Ep-ICD staining was observed in the normal esophageal tissue (panel C, I), while the ESCC showed intense nuclear and cytoplasmic immunostaining (panel C, II). The head and neck normal mucosa showed faint membrane Ep-ICD (panel D, I), while intense nuclear and cytoplasmic immunostaining was observed in HNSCC (panel D, II).

**Figure 2:**
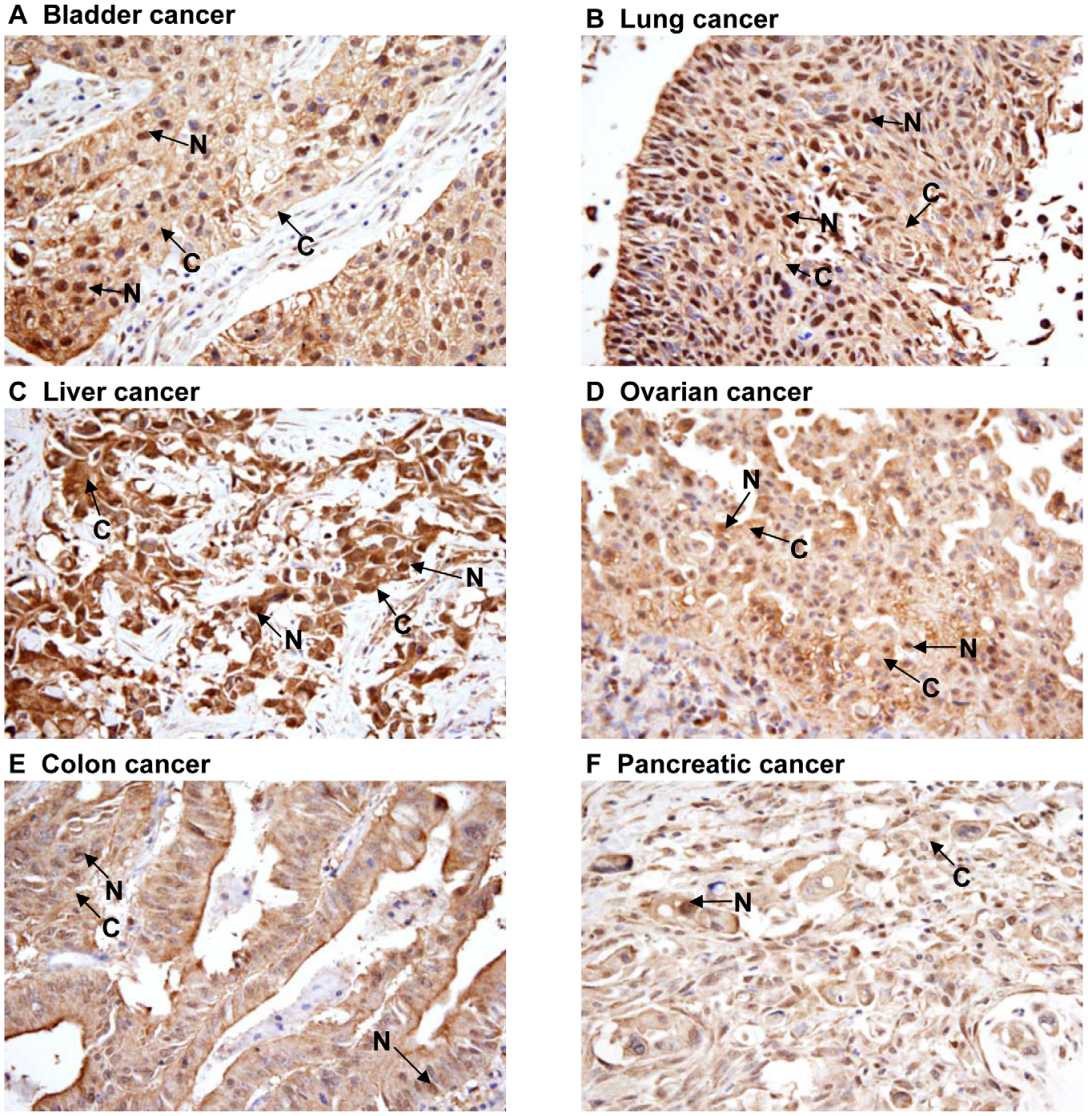
Immunohistochemical analysis of Ep-ICD expression in epithelial cancers. Increased nuclear and cytoplasmic immunoreactivity and reduced or absence of membrane staining of Ep-ICD was observed in cancers of the bladder (Panel A), lung (Panel B), liver (Panel C), ovary (Panel D), colon (Panel E), and pancreas (Panel F). Original magnification ×400.

**Figure 3:**
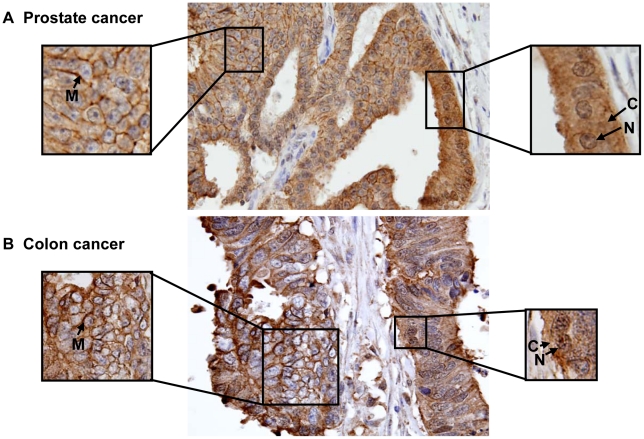
Immunohistochemical analysis of Ep-ICD expression in prostate and colon cancers. Membrane, cytoplasmic, and nuclear Ep-ICD expression in prostate cancer (A) and colon cancer (B). Some areas of the tissue section show predominant membrane and weak cytoplasmic but no nuclear localization of Ep-ICD (A, B - left square), while the other areas of the same tissue section show nuclear and cytoplasmic accumulation of Ep-ICD with absence of membranous Ep-ICD expression (A, B - right square). Original magnification ×400.

**Figure 4:**
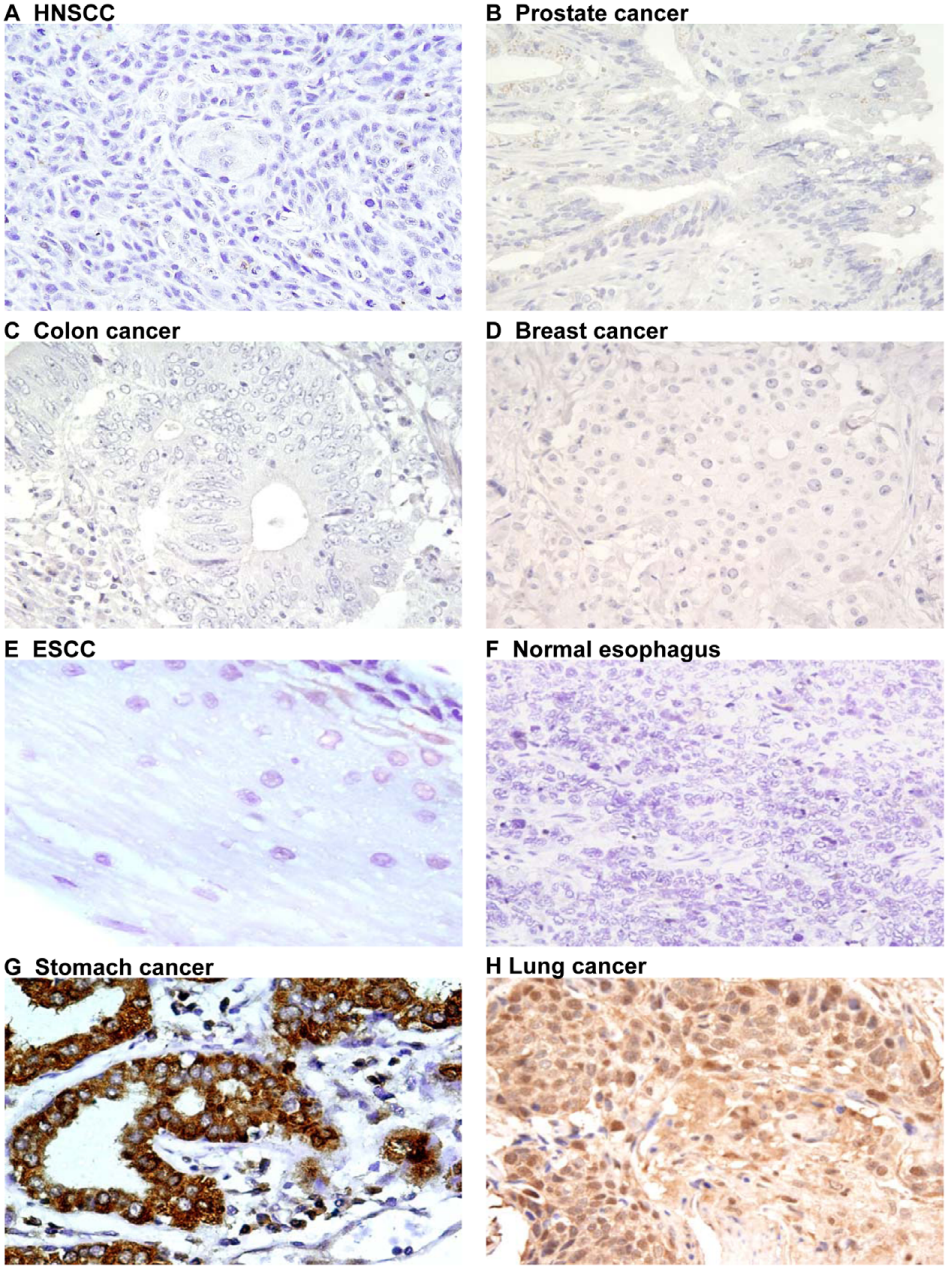
Ep-ICD immunohistochemical analysis in epithelial cancers control tissues. The negative and positive control photomicrographs are shown. Ep-ICD negative controls for HNSCC (A), prostate cancer (B), colon cancer (C), breast cancer (D), ESCC (E), and normal esophagus (F); panels G and H are positive controls for Ep-ICD staining. Original magnification ×400.

Increased nuclear and cytoplasmic immunoreactivity and reduced or absence of membrane staining of Ep-ICD was observed in cancers of the bladder (Figure 2, panel A), lung (B), liver (C), ovary (D), colon (E), and pancreas (F). Notably, membrane Ep-ICD staining was observed in some cases of each epithelial cancer analyzed. Representative photomicrographs of prostate cancer and colon cancer depicting heterogeneous Ep-ICD staining are shown in Figure 3 A, B. Some areas of the tissue section showed predominant membrane and weak cytoplasmic but no nuclear localization of Ep-ICD (Figure 3A, B - left square), while the other areas of the same tissue section showed nuclear and cytoplasmic accumulation of Ep-ICD with absence of membranous Ep-ICD expression (Figure 3A, B - right square). The negative and positive control photomicrographs are shown in Figure 4.

### Scatter Plot Analysis

**Figure 5:**
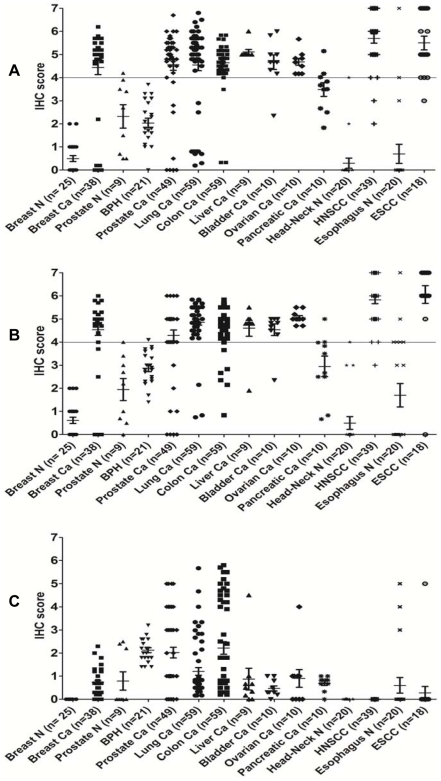
Scatter plot analysis of Ep-ICD membrane, cytoplasmic, and nuclear expression. Scatter plots showing distribution of total immunostaining scores determined by IHC of tissue sections of cancers of the breast (n = 38), prostate (n = 49), lung (n = 59), ovary (n = 10), colon (n = 59), bladder (n = 10), liver (n = 9), positively-staining HNSCCs (n = 39) and ESCCs (n = 19), and normal breast (n = 25), prostate (n = 9) and BPH (n = 21), normal esophageal (n = 20), and head and neck (n = 20) tissues. The vertical axis gives the total IHC score as described in the Methods. A cutoff of ≥4 was used to determine positivity. N, normal; Ca, cancer. A. Increased nuclear accumulation of Ep-ICD was observed in most of the epithelial cancers analyzed. B. Increased cytoplasmic accumulation of Ep-ICD was observed in almost all epithelial cancers analyzed. C. Membrane localization of Ep-ICD varied in the different cancer and normal tissue types examined.

The scatter plots in Figure 5A and 5B show the distribution of nuclear and cytoplasmic Ep-ICD immunostaining scores in all the epithelial cancer types analyzed. Nuclear Ep-ICD was observed in 39 of the 57 HNSCC tissues and in 18 of the 46 ESCC tissues. Thirty seven of these 39 HNSCC tissues examined and 17 of the 18 ESCC tissues showed IHC score ≥4. Cytoplasmic Ep-ICD staining was detected in 42 of the 57 (74%) HNSCC tissues examined and 32 of the 46 (70%) ESCC tissues. Breast cancer, prostate cancer, and positively-staining HNSCCs and ESCCs all exhibited notable elevations of both nuclear and cytoplasmic Ep-ICD compared to the normal tissues and prostate benign hyperplastic tissues analyzed. Lung, colon, liver, bladder, ovarian, and pancreatic cancer types each demonstrated prominent expression of nuclear and cytoplasmic Ep-ICD. The scatter plots in Figure 5C show the distribution of membrane Ep-ICD scores in all the epithelial cancer types analyzed.

### Immunohistochemical analysis of EpEx expression in human epithelial cancers

**Figure 6:**
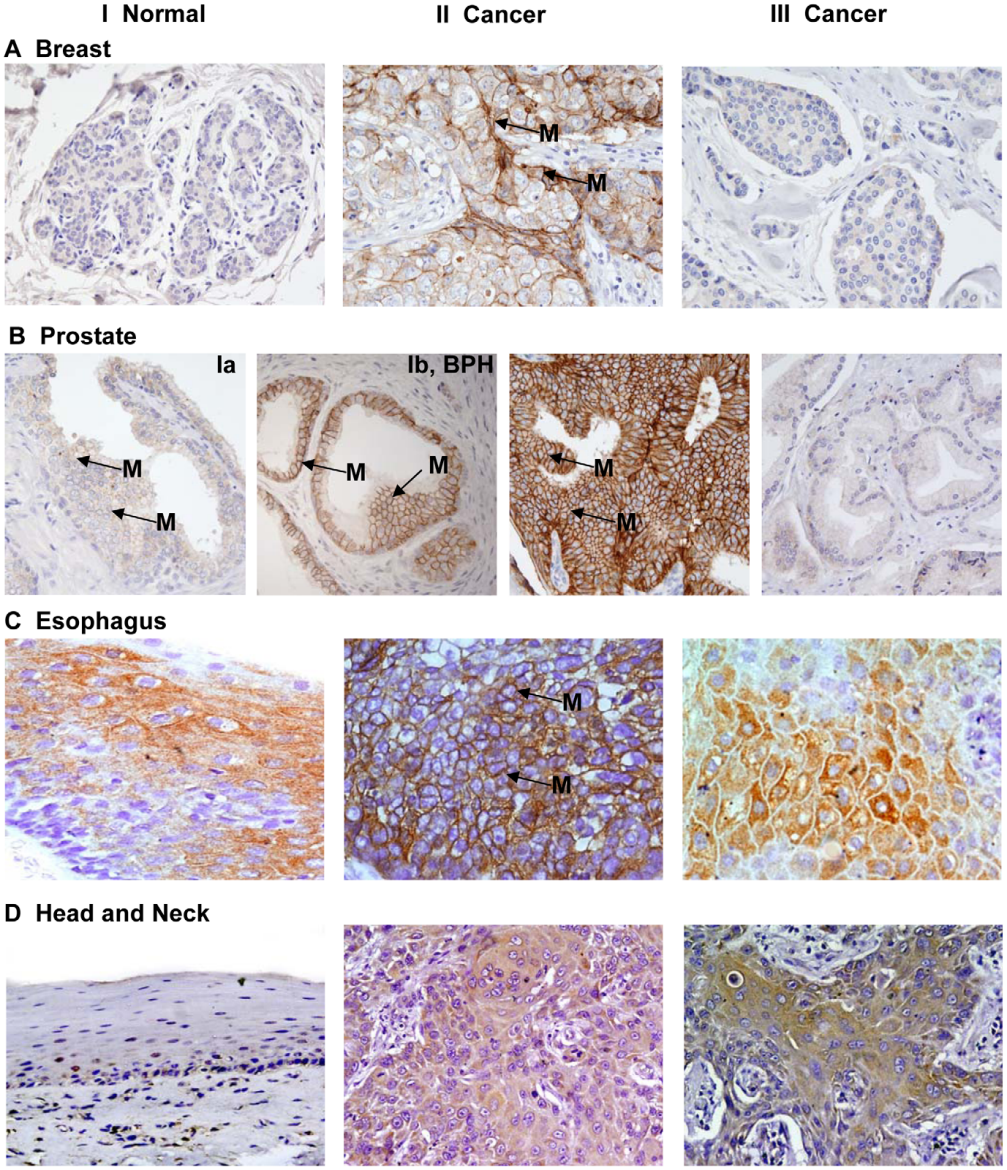
Immunohistochemical analysis of EpEx expression in epithelial cancers. The photomicrographs depict MOC31 stained membrane EpEx in epithelial cancers. The panel I shows low level of membrane EpEx expression in normal breast (A) and prostate (B, Ia), BPH (B, Ib), and normal esophagus (C) and head and neck (D) tissues. The corresponding cancer tissues depicting increased level of EpEx in the membrane are shown in panel II (A–D). In contrast many of the cancer tissues of each cancer type showed absence of membrane EpEx (panel III, A–D). Original magnification ×400.

**Figure 7:**
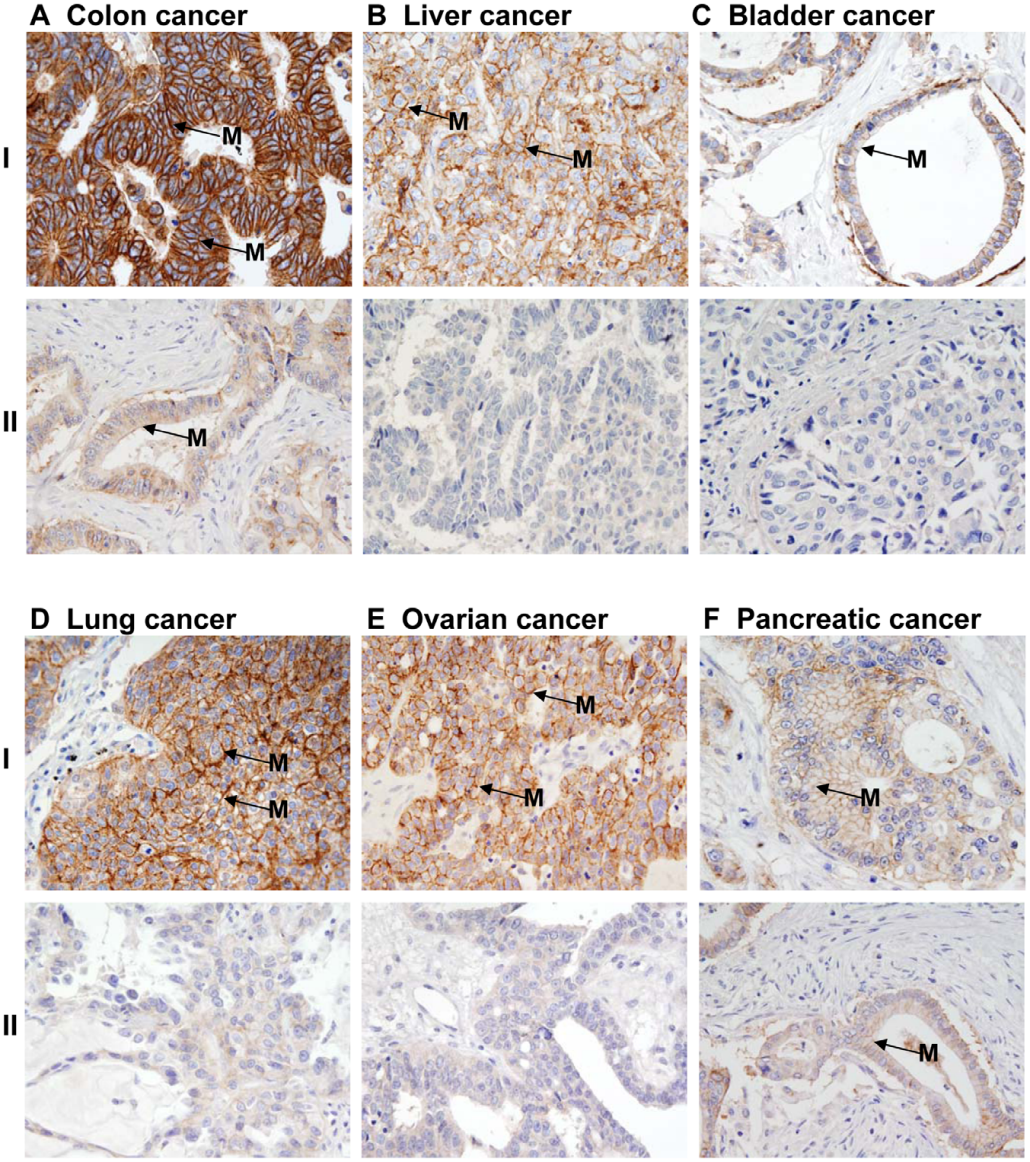
Immunohistochemical analysis of EpEx expression in epithelial cancers. Membrane EpEx expression was observed in all the epithelial cancers. Panel I shows intense membrane EpEx in colon cancer (A), liver (B), bladder (C), lung (D), ovarian (E) and pancreatic (F) cancer. The panels II A–F show reduced or absence of membrane EpEx in a subset of each of these epithelial cancers. Original magnification ×400.

Similar IHC analysis of EpEx expression in these human epithelial cancers was also carried out using MOC31, a specific antibody against the extracellular domain of EpCAM (EpEx) in all the ten epithelial cancers (Figure 6 and 7). Representative photomicrographs in Figure 6, panel I show low level of membrane EpEx expression in normal breast (A) and prostate (B, Ia), BPH (B, Ib), and normal esophagus (C) and head and neck (D) tissues. The corresponding cancer tissues depicting increased level of EpEx in the membrane are shown in Figure 6, panel II (A- breast; B-prostate; C- esophagus; and D- head and neck). In contrast, many of the cancer tissues of each cancer type showed absence of membrane EpEx; representative photomicrographs are shown in panel III (A–D). Figure 7, panel I shows intense membrane EpEx in colon cancer (A), liver (B), bladder (C), lung (D), ovary (E) and pancreatic (F) cancer. Figure 7 panel II (A–F) shows reduced or absence of membrane EpEx in a subset of each of these epithelial cancers. The positive and negative controls are shown in Supplementary Figure S1.

### Analysis of Ep-ICD and EpEx immunofluorescence in CX-1 colon cancer cell line

**Figure 8:**
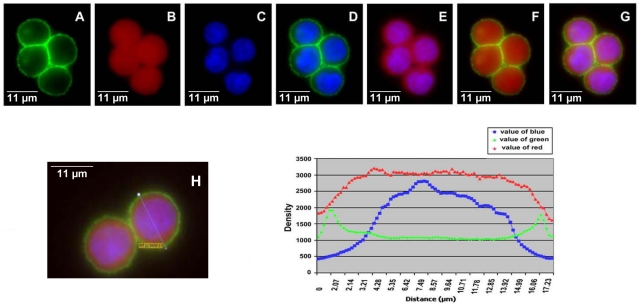
Fluorescence immunostaining in CX-1 cells with anti-EpEx (MOC31) and anti-Ep-ICD antibodies. Secondary antibodies are FITC-anti-mouse (green) and TRITC-anti-rabbit (red). A) EpEx; B) Ep-ICD; C) DAPI; D) EpEx and DAPI (A&C merged); E) Ep-ICD and DAPI (B & C merged); F) EpEx and Ep-ICD (A & B merged); G) EpEx, Ep-ICD, and DAPI (A, B, C merged); I) Measurement of the density of three colors across the line in the cells in H.

In CX-1, an aggressive colon cancer cell line, EpEx and Ep-ICD were both detected in the plasma membrane (Figure 8). Intense membrane expression was observed at cell-cell junctions with both EpEx and Ep-ICD domain specific antibodies (Figure 8 - A, D, F, and G). In addition, accumulation of Ep-ICD was observed in the cytoplasm and nuclei of cancer cells, but no EpEx accumulation was found in the nuclei (Figure 8 - C, E, G). The quantitative fluorescence signal scan of EpEx and Ep-ICD clearly supports these observations (Figure 8H).

### ROC Curve Analysis

**Figure 9:**
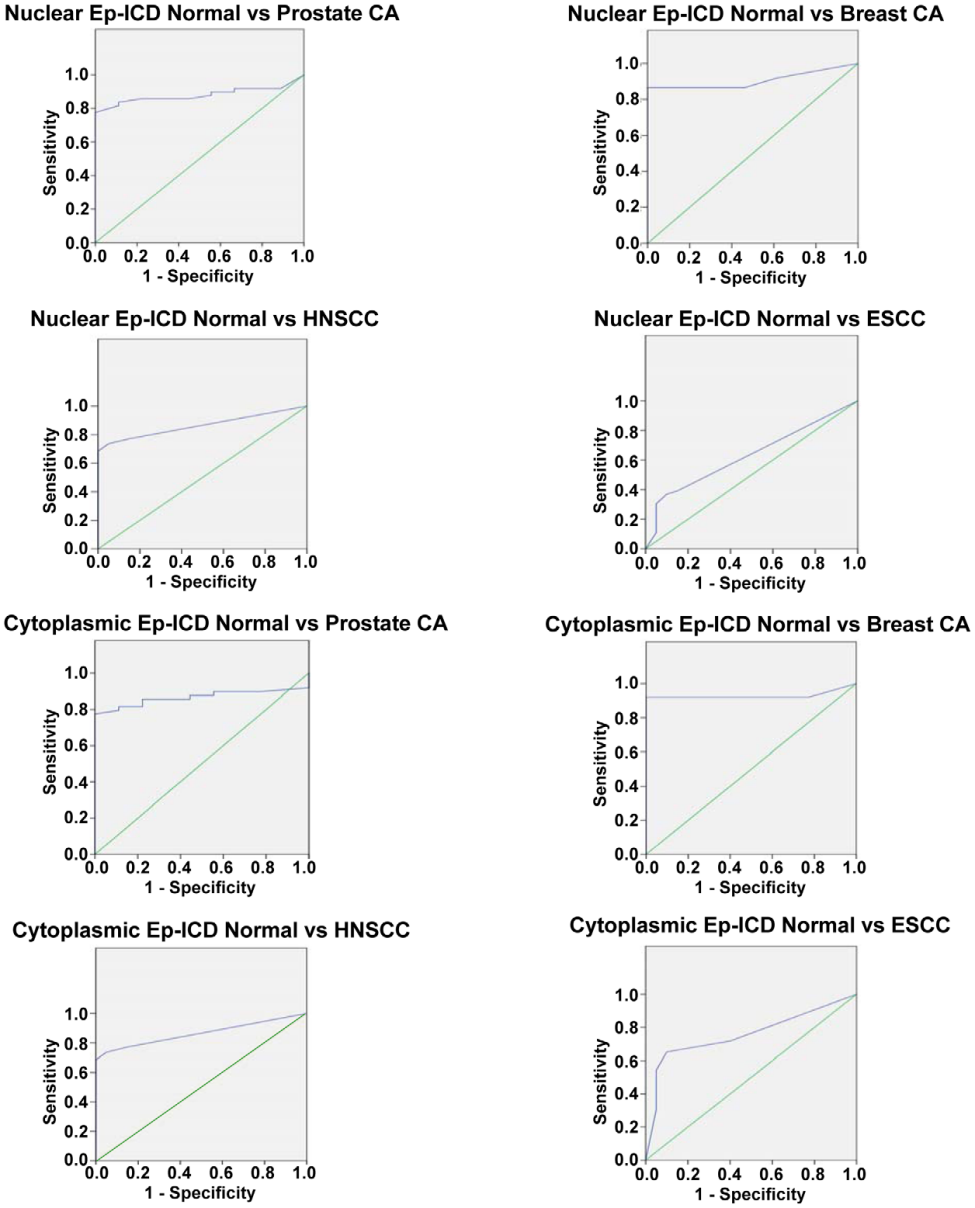
ROC curves of nuclear and cytoplasmic Ep-ICD in prostate cancer, breast cancer, HNSCC, and ESCC. ROC curves describing the relationship between sensitivities and 1-specificities of nuclear and cytoplasmic Ep-ICD expression in these epithelial cancers. The vertical axis of each curve indicates sensitivity and the horizontal axis indicates the 1-specificity. The sensitivity, specificity, and AUC values for the cancers are summarized in Table 2.

ROC curves were generated for nuclear and cytoplasmic Ep-ICD in HNSCC, ESCC, prostate, and breast cancers (Figure 9) and their results are summarized in Table 2. Based on this analysis, a cutoff of ≥4 was used to determine nuclear and cytoplasmic positivity. Analysis revealed that nuclear Ep-ICD accumulation distinguished prostate cancers and breast cancers from normal tissues with 82% sensitivity and with a specificity of 100% for breast and 78% for prostate. In HNSCCs, nuclear Ep-ICD was able to distinguish cancers from normal tissues with 65% sensitivity and 95% specificity, while in ESCCs, nuclear Ep-ICD showed 37% sensitivity and 90% specificity. The AUC values were found to be 0.905 for breast cancer, 0.867 for prostate cancer, 0.822 for HNSCC, and 0.630 for ESCC. Cytoplasmic Ep-ICD expression in breast and prostate cancer had a sensitivity of 82% and 84% respectively, with a specificity of 100% for breast and 89% for prostate. In HNSCC, cytoplasmic Ep-ICD expression had a sensitivity of 74% and a specificity of 95%, while cytoplasmic Ep-ICD had sensitivity and specificity of 70% each in ESCCs. The AUC values for cytoplasmic Ep-ICD were 0.928 in breast cancer, 0.880 in prostate cancer, 0.864 in HNSCC, and 0.758 in ESCC.

**Table 2:** Biomarker Analysis of Nuclear and Cytoplasmic Ep-ICD Expression in Epithelial Cancers.

Ep-ICD Nuclear IHC Scores	AUC	Sensitivity (%)	Specificity (%)	PPV (%)	NPV (%)	Asymptotic Sig.
**Prostate Cancer vs. Normal**	0.867	82	78	95	44	0.001
**Breast Cancer vs. Normal**	0.905	82	100	100	78	0.000
**HNSCC vs. Normal**	0.822	65	95	97	49	0.000
**ESCC vs. Normal**	0.630	37	90	90	38	0.001

Ep-ICD Cytoplasmic IHC Scores	AUC	Sensitivity (%)	Specificity (%)	PPV (%)	NPV (%)	Asymptotic Sig.
**Prostate Cancer vs. Normal**	0.880	82	89	98	47	0.000
**Breast Cancer vs. Normal**	0.928	84	100	100	81	0.000
**HNSCC vs. Normal**	0.864	74	95	98	56	0.000
**ESCC vs. Normal**	0.758	70	70	84	50	0.001

## Discussion

The current study highlights the frequent occurrence of nuclear and cytoplasmic accumulation of Ep-ICD in ten epithelial cancer types. The normal epithelia of the breast, prostate and esophageal tissues analyzed showed distinct membrane Ep-ICD localization. Importantly, reduced or absence of membranous Ep-ICD was observed in cancers of breast, prostate, head and neck and esophagus, paralleled by marked Ep-ICD accumulation in the nucleus and cytoplasm of the respective tumor cells. However, a subset of tumors of each of these ten cancer types showed high Ep-ICD membrane expression that correlated with high EpEx expression on the membrane detected using an Ep-CAM external domain (EpEx) specific antibody (MOC31), suggesting increased expression of the full length protein as detected by Ep-ICD and EpEx specific antibodies. Notably, immunohistochemical analysis of epithelial cancers using MOC31 showed increased EpEx membrane expression in comparison with the normal tissues in some tumors, while the others showed reduced or absence of membrane EpEx, supporting our observations with Ep-ICD domain specific antibodies. It is worthwhile to point out that cancers showing reduced or absence of membrane EpEx or Ep-ICD had markedly increased Ep-ICD in the cytoplasm and nuclei of such tumor cells. Notably, EpEx was not detected in any of the nuclei of tumor cells in any of the cancers analyzed. Taken together, these observations favor increased expression and increased shedding of EpEx by tumor cells in a subset of all epithelial tumors studied. The dual labeling immunofluorescence studies in an aggressive colon cancer cell line also clearly demonstrated localization of EpEx and Ep-ICD on the membrane, especially at the cell- cell contacts, with increased cytoplasmic and nuclear Ep-ICD expression. These observations favor increased expression and shedding of EpEx in cancer cells. Furthermore, we found increases in nuclear and cytoplasmic Ep-ICD in lung, colon, liver, bladder, ovarian, and pancreatic cancers as well as reduced or absence of membrane EpEx in these cancers. However, some tumors of each of the ten cancer type analyzed showed intense membrane EpEx expression. It is important to note that our observations are in accordance with the summary of EpCAM data in the Human Protein Atlas database (http://www.proteinatlas.org/) using EpEx-specific antibodies that reported variable expression of EpCAM in different human cancers. The novelty of our study is the use of Ep-ICD specific antibodies for IHC analysis of Ep-ICD expression in ten epithelial cancer types and the demonstration of nuclear and cytoplasmic Ep-ICD expression with the paralleled reduction or absence of membranous Ep-ICD in subsets of each of these cancer types.

Except for a previous report on Ep-ICD in colon cancer [24] and our study on thyroid cancer [28], this study represents the first report on Ep-ICD occurrence in cancers of breast, prostate, head and neck, esophagus, ovary, lung, urinary bladder, pancreas and liver. The diagnostic utility of nuclear and cytoplasmic Ep-ICD expression was validated by the vast difference in their observed expression levels between normal tissue and the breast and prostate cancers. Nuclear Ep-ICD expression was able to distinguish normal tissue from breast and prostate cancers with AUC values of 0.905 and 0.867 respectively and with 82% sensitivity and 100% specificity for breast cancers and 82% sensitivity and 78% specificity for prostate cancers. For the head and neck cancers nuclear Ep-ICD had an AUC value of 0.822, with 65% sensitivity and 95% specificity and cytoplasmic Ep-ICD also showed similar diagnostic performance. The esophageal cancers showed an AUC value of 0.758, with 70% sensitivity and 70% specificity for cytoplasmic Ep-ICD, though lower frequency of nuclear positivity was observed in these cancers. The high sensitivity, specificity, and AUC value of nuclear and cytoplasmic Ep-ICD expression in prostate, breast and head and neck cancers underscore its diagnostic utility in these malignancies. Furthermore, high frequencies of nuclear and cytoplasmic Ep-ICD were observed in lung and colon cancers, though the non-availability of normal tissues precluded the determination of ROC analysis for these cancers. High nuclear and cytoplasmic Ep-ICD immunopositivity was observed in cancers of the liver, bladder and ovary; the small number of cases analyzed nevertheless establishes the occurrence of Ep-ICD in these cancers, although further studies using larger cohorts are needed to determine the diagnostic significance of Ep-ICD versus EpEx subcellular accumulation in these epithelial cancers.

Quantitative real-time-PCR analyses of EpCAM expression in tumors and in circulating tumor cells from patients with many of these epithelial cancers reported increased levels of EpCAM transcripts that prospectively demonstrated prognostic significance for breast, colorectal and prostate cancer patients with advanced disease [30], [31], [32], [33], [34], [35]. A recent study by Punnoose et al., [34] reported EpCAM-based circulating tumor cell count to be higher in ER+ breast cancer patients in comparison to HER2+ and triple negative patients. The low EpCAM expression in HER2+ and triple negative breast cancers was attributed to a more mesenchymal phenotype. Another report on EpCAM expression in surgical specimens from esophageal cancer patients (n = 138), using real-time RT-PCR, IHC and ELISA showed that the mean expression level of EpCAM mRNA in tumor tissues was significantly higher than that in corresponding normal tissues (P<0.0001) [36]. The survival rates of patients with high EpCAM expression in tumors and in the peripheral vein were significantly higher than those for patients having low EpCAM expression. However, further studies will be needed to determine if low EpCAM expression in these tumors can be attributed to its regulated intramembrane proteolysis. The prognostic relevance of Ep-ICD versus EpEx subcellular expression in epithelial cancers remains to be examined in future studies. Notably, our earlier report in thyroid cancers demonstrated a strong association between nuclear accumulation of Ep-ICD and a reduced membranous EpEx localization as well as shortened overall survival, suggesting the potential application of the relative subcellular expression of Ep-ICD versus EpEx as prognostic markers for aggressive thyroid cancers. Taken together with the findings of the current study, our observations strongly support that the accumulation of nuclear and /or cytoplasmic Ep-ICD could favour its clinical application as a putative prognostic marker in several epithelial cancers surveyed in this report. It is therefore important to examine in future studies, the potential of nuclear and cytoplasmic Ep-ICD versus membrane EpEx accumulation as prognostic indicator(s) of aggressive subtypes of many other epithelial cancers anticipated to have shortened life expectancy and the need for more aggressive therapies. Such studies conducted on large cohorts will be essential in understanding the clinical significance of nuclear and cytoplasmic Ep-ICD accumulation and thereby lead to the translation of such findings to novel diagnostic and treatment strategies.

Previous strategies for EpCAM-based targeted therapies focused on monoclonal antibodies to EpEx including the humanized antibodies that are in phase III trials [37]. Our current findings in a large spectrum of epithelial cancers suggest that absence of membranous EpEx and the increase in nuclear and cytoplasmic accumulation of Ep-ICD may explain the limited efficacy of EpEx-directed targeted therapies in some of these epithelial cancers, including breast cancer [22]. Ep-ICD directed targeted therapies are likely to be effective in the management of cancers showing enhanced nuclear and cytoplasmic accumulation of Ep-ICD. The future application of targeted immunotherapy may thereby require improved individualized immunodiagnostic selection of patients based on their relative expression of Ep-ICD versus EpEx in the tumors of each affected patient. This therapeutic goal may be achieved by developing robust high throughput assays on clinical tumor tissue samples for these two EpCAM components to serve as a guide to target monoclonal antibody therapy. This strategy offers the hope that the results for many epithelial cancers can be enhanced by determining the appropriate EpCAM target.

### Conclusions

In conclusion, we provide new evidence of increased nuclear and cytoplasmic Ep-ICD accumulation and a variable increase or absence of membrane EpEx expression in a wide variety of epithelial cancers. The high frequency of nuclear and cytoplasmic Ep-ICD accumulation observed in most epithelial cancers studied to-date strongly suggests that Ep-ICD may play an important role in epithelial-derived cancers. The high AUC value of Ep-ICD and its ability to distinguish cancers from normal tissues with high sensitivity and specificity, suggests it may serve as a putative biomarker for increased oncogenesis. Future diagnostic, prognostic and targeted therapies may be improved by more refined selection of patients based on Ep-ICD versus EpEx expression in tumors.

## Supporting Information

Figure S1 EpEx immunohistochemical analysis in epithelial cancers control tissues. The negative control photomicrographs are shown. HNSCC (A), prostate cancer (B), colon cancer (C), breast cancer (D), ESCC (E) and normal esophagus (F); panels G and H are positive controls for EpEx staining. Original magnification ×400.
(6.53 MB TIF)

Table S1 Ep-ICD Accumulation and Clinicial Parameters of Prostate Cancer Patients. AC: adenocarcinoma; BPH: benign prostate hyperplasia.
(0.17 MB PDF)

Table S2 Ep-ICD Accumulation and Clinical Parameters of Lung Cancer Patients. Abbreviations: AC: adenocarcinoma; BAC: bronchioalveolar carcinoma; M: mucinous; MD: moderately-differentiated; NM = non-mucinous; PD: poorly-differentiated; WD = well-differentiated.
(0.11 MB PDF)

Table S3 Ep-ICD Accumulation and Clinical Parameters of Colon Cancer Patients. Abbreviations: AC: adenocarcinoma; MC: mucinous carcinoma; MD: moderately-differentiated; PD: poorly-differentiated; WD = well-differentiated.
(0.08 MB PDF)

Table S4 Ep-ICD Accumulation and Clinical Parameters of Breast Cancer Patients. Abbreviations: IDC: infiltrating duct carcinoma.
(0.17 MB PDF)

Table S5 Ep-ICD Accumulation and Clinical Parameters of Liver Cancer Patients.
(0.02 MB PDF)

Table S6 Ep-ICD Accumulation and Clinical Parameters of Bladder Cancer Patients. Abbreviations: TCC: transitional cell carcinoma.
(0.02 MB PDF)

Table S7 Ep-ICD Accumulation and Clinical Parameters of Ovarian Cancer Patients. Abbreviations: MAC: mucinous adenocarcinoma; MD: moderately differentiated; PD: poorly differentiated; SAC: serous adenocarcinoma.
(0.02 MB PDF)

Table S8 Ep-ICD Accumulation and Clinical Parameters of Pancreatic Cancer Patients. Abbreviations: MDDAC: moderately differentiated ductal adenocarcinoma; PDDAC: poorly differentiated ductal adenocarcinoma.
(0.02 MB PDF)

Table S9 Ep-ICD Accumulation and Clinical Parameters of HNSCC Patients. Abbreviations: MD: moderately differentiated; PD: poorly differentiated; SCC: squamous cell carcinoma; WD: well differentiated.
(0.08 MB PDF)

Table S10 Ep-ICD Accumulation and Clinical Parameters of ESCC Patients. Abbreviations: AC: adenocarcinoma; MD: moderately differentiated; PD: poorly differentiated; SCC: squamous cell carcinoma; WD: well differentiated.
(0.10 MB PDF)
