# Supplementary figures and images for: Nuclear and Cytoplasmic Accumulation of Ep-ICD Is Frequently Detected in Human Epithelial Cancers
Source: PLoS One. 2010 Nov 30;5(11):e14130. doi: 10.1371/journal.pone.0014130 (PMC2994724; doi:10.1371/journal.pone.0014130)

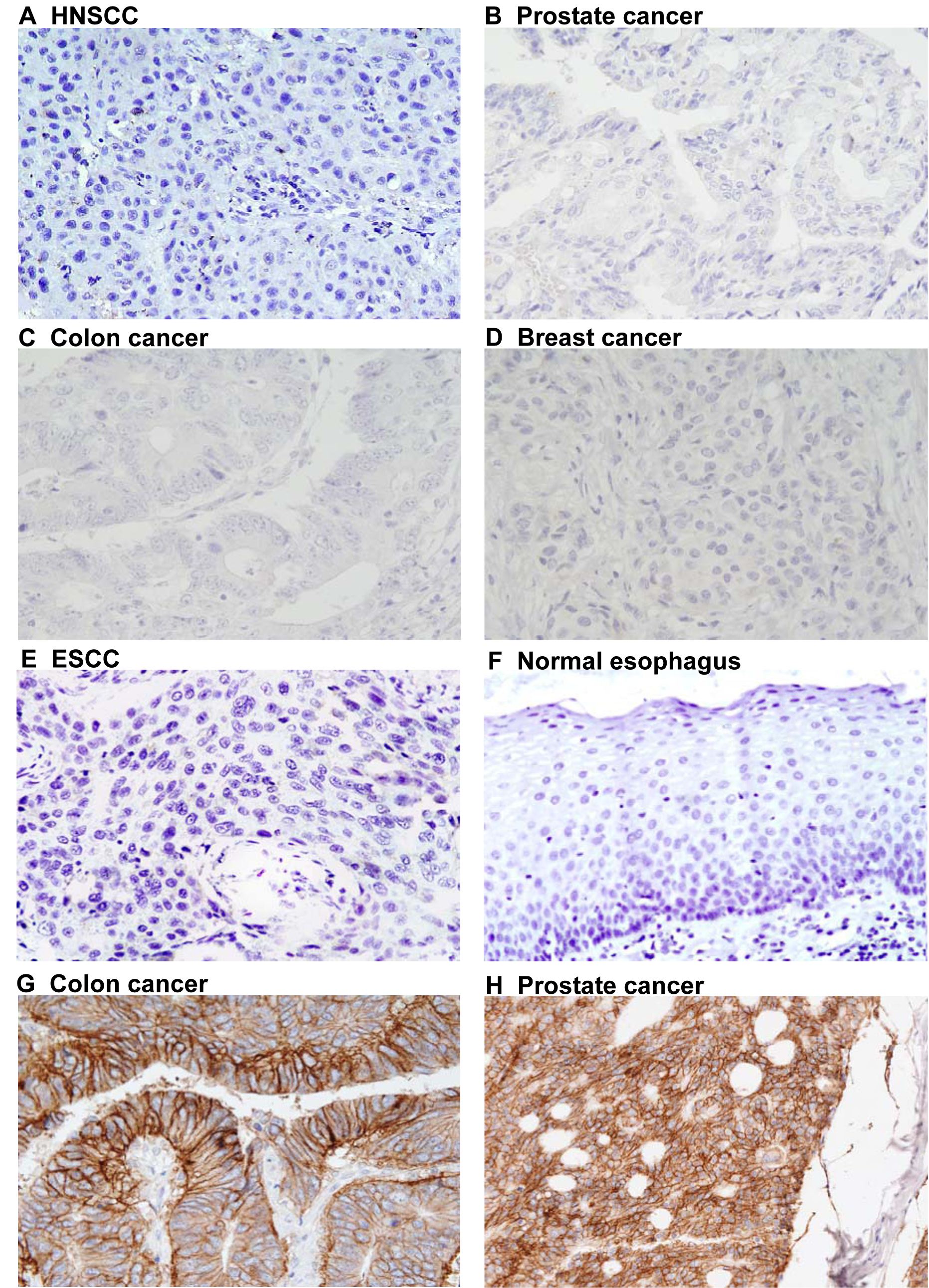

Supplement: Figure S1 — EpEx immunohistochemical analysis in epithelial cancers control tissues. The negative control photomicrographs are shown. HNSCC (A), prostate cancer (B), colon cancer (C), breast cancer (D), ESCC (E) and normal esophagus (F); panels G and H are positive controls for EpEx staining. Original magnification ×400. (6.53 MB TIF) [file pone.0014130.s001.tif]
