# Supplementary material for: Nuclear and Cytoplasmic Accumulation of Ep-ICD Is Frequently Detected in Human Epithelial Cancers
Source: PLoS One. 2010 Nov 30;5(11):e14130. doi: 10.1371/journal.pone.0014130 (PMC2994724; doi:10.1371/journal.pone.0014130)
Supplement: Table S1 — Ep-ICD Accumulation and Clinicial Parameters of Prostate Cancer Patients. AC: adenocarcinoma; BPH: benign prostate hyperplasia. (0.17 MB PDF) [file pone.0014130.s002.pdf]

Supplementary Table S1 - Ep-ICD Accumulation and Clinicial Parameters of Prostate Cancer Patients

n	Age	Sex	Organ	Diagnosis	Gleason score	pTNM	Stage	Tissue type*	PSA (ng/ml)	Residual tumor	Follow-up months	Follow-up result	Ep-ICD Nucleus	Ep-ICD Cytoplasm	Ep-ICD Membrane
1	60	M	Prostate	AC	9	T3bN0M0	III	6	11.2	R0	60	alive	2.5	6	5
2	64	M	Prostate	AC	7	T2cN0M0	II	6	30	R0	60	alive	4.8	5.3	0.7
3	71	M	Prostate	AC	9	T3bN0M0	III	6	60.4	R1	55	alive	5.7	5.3	0.7
4	64	M	Prostate	AC	10	T3aN0M0	III	6	7.4	R1	47	alive	5.3	2.2	0
5	59	M	Prostate	AC	9	T3bN0M0	III	6	9.8	R1	44	alive	3.8	3.8	1
6	65	M	Prostate	AC	8	T4N0M0	IV	6	34.9	R1	43	alive	0.5	5.3	5.3
7	73	M	Prostate	AC	7	T2cN0M0	II	6	48.1	R1	42	alive	4.7	0.8	0.2
8	69	M	Prostate	AC	7	T2cN0M0	II	6	10.6	R0	42	alive	5	5.2	4
9	62	M	Prostate	AC	7	T2cN0M0	II	6	37.3	R1	39	alive	5.7	4.8	0.8
10	66	M	Prostate	AC	9	T3bN0M0	III	6	1.2	R1	39	alive	0	0	0
11	60	M	Prostate	AC	9	T3bN0M0	III	6	40	R1	39	alive	5.7	5.2	3.2
12	66	M	Prostate	AC	7	T3aN0M0	III	6	8.4	R1	39	alive	5.7	4.7	3
13	70	M	Prostate	AC	7	T4N0M0	IV	6	7	R1	37	alive	6.7	5	0.8

14	65	M	Prostate	AC	9	T3bN0M0	III	6	17.5	R1	23	dead	5.5	5.2	4.7
15	67	M	Prostate	AC	9	T3bN1M0	IV	6	13.1	R1	34	alive	5	4.7	0.8
16	69	M	Prostate	AC	7	T3bN0M0	III	6	1.1	R1	33	alive	3.8	4.2	3.8
17	63	M	Prostate	AC	9	T3aN1M0	IV	6	11.8	R1	33	alive	0	0	0
18	69	M	Prostate	AC	7	T3aN0M0	III	6	17.6	R1	27	alive	5.8	4.8	3.3
19	70	M	Prostate	AC	7	T3aN0M0	III	6	9	R1	26	alive	5	4.8	0.7
20	58	M	Prostate	AC	9	T3bN0M0	III	6	5.8	R0	26	alive	4.8	5.3	0.8
21	58	M	Prostate	AC	7	T3bN0M0	III	6	15.8	R1	24	alive	5.3	5.8	2.3
22	71	M	Prostate	AC	7	T2cN0M0	II	6	31.4	R1	24	alive	5.3	4.8	1.7
23	70	M	Prostate	AC	7	T3bN0M0	III	6	14.4	R1	19	alive	5.5	5.8	0.8
24	59	M	Prostate	AC	6	T2bN0M0	II	6	18.3	R0	18	alive	6	4.7	4
25	63	M	Prostate	AC	9	T3bN0M0	III	6	16.6	R1	17	alive	5.5	4.8	3.8
26	72	M	Prostate	AC	9	T3bN0M0	III	6	.	R1	16	alive	0	0	0
27	66	M	Prostate	AC	8	T3bN0M0	III	6	10.8	R1	17	dead	6	5.2	0.5
28	70	M	Prostate	AC	6	T3bN0M0	III	6	10.8	R1	16	alive	5.2	5.2	0.8
29	70	M	Prostate	AC	7	T2cN0M0	II	6	.	R0	15	alive	5.7	4.8	2.9

30	68	M	Prostate	AC	8	T3bN0M0	III	6	26.9	R1	15	alive	5.7	4.8	5
31	63	M	Prostate	AC	10	T3bN0M0	III	6	.	R1	15	alive	5.3	5.7	0.7
32	57	M	Prostate	AC	7	T3bN0M0	III	6	25	R1	15	alive	5.2	5.2	4.8
33	72	M	Prostate	AC	8	T2cN0M0	II	6	16.8	R1	15	alive	5.2	4	3.5
34	70	M	Prostate	AC	8	T3bN0M0	III	6	0.5	R1	15	alive	2.8	1.3	0.8
35	75	M	Prostate	AC	9	T3bN0M0	III	6	98	R1	15	alive	5.5	5.3	2.7
36	62	M	Prostate	AC	9	T3bN0M0	III	6	.	R1	15	alive	4.5	5	4
37	63	M	Prostate	AC	9	T3bN0M0	III	6	91	R1	14	alive	5.5	5	1
38	53	M	Prostate	AC	9	T3bN0M0	III	6	161	R1	17	dead	4.8	5	4
39	63	M	Prostate	AC	8	T3bN0M0	III	6	13	R1	13	alive	5.3	4.8	1.2
40	44	M	Prostate	AC	7	T3bN0M0	III	6	.	R1	11	alive	5	4.8	0.8
41	85	M	Prostate	AC		TxN0M1	IV	6	.	.	.	.	4.7	3.4	2.9
42	79	M	Prostate	AC		T2aN0M1	IV	6	.	.	.	.	6	4	1
43	53	M	Prostate	AC		T2aN0M1	IV	6	.	.	.	.	4.2	4.5	0.5
44	68	M	Prostate	AC		T2bN0M0	II	6	.	.	.	.	0	0	0
45	82	M	Prostate	AC		T2N0M1	IV	6	.	.	.	.	5	4.3	0.5

46	76	M	Prostate	AC		T3aN0M0	III	6	.	.	.	.	4	4.3	1.8
47	56	M	Prostate	AC		T2cN1M0	IV	6	.	.	.	.	4.9	5	0.8
48	66	M	Prostate	AC		T4N0M0	IV	6	.	.	.	.	4.5	5	0.8
49	54	M	Prostate	AC		T1aN0M0	II	6	.	.	.	.	4.8	4.7	0.8
50	69	M	Prostate	normal	.	.	.	3	.	.	.	.	4.2	2.7	2.4
51	62	M	Prostate	normal	.	.	.	3	.	.	.	.	3.9	4	0
52	66	M	Prostate	normal	.	.	.	3	.	.	.	.	4	3.4	2.5
53	65	M	Prostate	normal	.	.	.	3	.	.	.	.	3.4	3	0
54	69	M	Prostate	normal	.	.	.	3	.	.	.	.	1.5	1	0
55	70	M	Prostate	normal	.	.	.	3	.	.	.	.	0.5	0.5	0
56	70	M	Prostate	normal	.	.	.	3	.	.	.	.	0.5	0	0
57	63	M	Prostate	normal	.	.	.	3	.	.	.	.	0.7	0.7	0
58	44	M	Prostate	normal	.	.	.	3	.	.	.	.	2.7	2.2	2.2
59	59	M	Prostate	BPH	.	.	.	.	.	.	.	.	3.3	2.7	2.2
60	55	M	Prostate	BPH	.	.	.	.	.	.	.	.	1.8	3.8	3.2
61	66	M	Prostate	BPH	.	.	.	.	.	.	.	.	0	2.8	2

62	74	M	Prostate	BPH	.	.	.	.	.	.	.	.	3.3	4.1	1.8
63	74	M	Prostate	BPH	.	.	.	.	.	.	.	.	3.1	3.3	1.6
64	64	M	Prostate	BPH	.	.	.	.	.	.	.	.	1.8	2.5	1.8
65	31	M	Prostate	BPH	.	.	.	.	.	.	.	.	1.6	3.7	2
66	58	M	Prostate	BPH	.	.	.	.	.	.	.	.	2.5	2.9	2.2
67	70	M	Prostate	BPH	.	.	.	.	.	.	.	.	1	2.3	1.4
68	64	M	Prostate	BPH	.	.	.	.	.	.	.	.	1.1	2.2	2.8
69	67	M	Prostate	BPH	.	.	.	.	.	.	.	.	1	3.4	2.6
70	79	M	Prostate	BPH	.	.	.	.	.	.	.	.	1	2.4	1.8
71	79	M	Prostate	BPH	.	.	.	.	.	.	.	.	1.7	3	1.4
72	59	M	Prostate	BPH	.	.	.	.	.	.	.	.	2.1	1.4	2.6
73	61	M	Prostate	BPH	.	.	.	.	.	.	.	.	2.9	3.7	2.4
74	55	M	Prostate	BPH	.	.	.	.	.	.	.	.	3.1	3.6	2.2
75	58	M	Prostate	BPH	.	.	.	.	.	.	.	.	2.4	1.7	2.2
76	74	M	Prostate	BPH	.	.	.	.	.	.	.	.	2.3	2.8	1.4
77	64	M	Prostate	BPH	.	.	.	.	.	.	.	.	3.7	2.8	1.8

78	67	M	Prostate	BPH	.	.	.	.	.	.	.	.	1.4	3	2.6
79	64	M	Prostate	BPH	.	.	.	.	.	.	.	.	1.6	2.3	2.6
