# Supplementary material for: Nuclear and Cytoplasmic Accumulation of Ep-ICD Is Frequently Detected in Human Epithelial Cancers
Source: PLoS One. 2010 Nov 30;5(11):e14130. doi: 10.1371/journal.pone.0014130 (PMC2994724; doi:10.1371/journal.pone.0014130)
Supplement: Table S2 — Ep-ICD Accumulation and Clinical Parameters of Lung Cancer Patients. Abbreviations: AC: adenocarcinoma; BAC: bronchioalveolar carcinoma; M: mucinous; MD: moderately-differentiated; NM = non-mucinous; PD: poorly-differentiated; WD = well-differentiated. (0.11 MB PDF) [file pone.0014130.s003.pdf]

Supplementary Table S2 - Ep-ICD Accumulation and Clinical Parameters of Lung Cancer Patients

n	Age	Sex	Organ	Diagnosis	LN*	pTNM	Stage	Follow-up months	Follow-up result	Ep-ICD Nucleus	Ep-ICD Cytoplasm	Ep-ICD Membrane
1	56	M	Lung	WDSCC	5/43	T2N1M0	IIB	46	dead	5.5	4.2	3.3
2	47	F	Lung	MDAC	0/30	T2N0M0	IB	0	lost	0.7	5.3	2.7
3	34	F	Lung	WDAC	18/30	T2N2M0	IIIA	24	dead	0.8	4.8	2.2
4	69	F	Lung	WDAC	0/15	T2N0M0	IB	118	alive	5.3	5.7	2.5
5	59	M	Lung	MDSCC	1/15	T2N1M0	IIB	117	alive	5.4	5.7	0.7
6	62	M	Lung	MDSCC	0/8	T2N0M0	IB	22	dead	0.8	4.8	1.0
7	42	M	Lung	BAC, NM	7/17	T4N2M0	IIIB	16	dead	4.8	5.5	0.3
8	64	M	Lung	WDAC	0/39	T2N0M0	IB	116	alive	4.8	4.8	2.0
9	64	M	Lung	WDSCC	0/15	T2N0M0	IB	15	dead	0.8	4.7	2.8
10	68	M	Lung	MDSCC	0/16	T3N0M0	IIB	6	dead	0.3	4.7	0.8
11	54	M	Lung	carcinosarcoma	0/6	T3N0M0	IIB	15	dead	5.3	5.5	0.8
12	73	M	Lung	large cell neuroendocrine carcinoma	0/7	T2N0M0	IB	10	dead	6.0	5.7	0.8
13	66	M	Lung	WDSCC	2/20	T3N2M0	IIIA	7	dead	5.8	4.2	0.2

14	59	F	Lung	large cell carcinoma	0/9	T2N0M0	IB	4	dead	5.7	4.3	0.3
15	59	M	Lung	WDSCC	4/61	T2N2M0	IIIA	10	dead	5.8	5.0	2.8
16	65	M	Lung	BAC, NM	0/8	T2N0M0	IB	110	alive	4.8	5.5	0.8
17	64	M	Lung	BAC, M	0/8	T2N0M0	IB	112	alive	5.0	2.2	5.7
18	61	M	Lung	MDSCC	0/16	T2N0M0	IB	109	alive	5.8	5.8	4.7
19	69	F	Lung	MDSCC	0/34	T2N0M0	IB	52	dead	5.5	5.7	0.8
20	64	M	Lung	WDSCC	0/17	T2N0M0	IB	12	dead	6.0	5.5	0.8
21	74	M	Lung	WDSCC	1/46	T2N1M0	IIB	18	dead	4.5	5.2	0.8
22	53	F	Lung	SCC, spindle cell	0/10	T2N0M0	IB	92	dead	5.2	5.7	0.7
23	61	M	Lung	WDSCC	1/43	T2N1M0	IIB	46	dead	5.5	5.5	0.7
24	59	F	Lung	BAC, M	0/24	T2N0M0	IB	41	dead	2.5	0.8	0.8
25	58	M	Lung	large cell carcinoma	0/14	T2N0M0	IB	100	alive	5.7	5.8	0.7
26	77	M	Lung	MDSCC	5/18	T2N2M0	IIIA	20	dead	0.8	4.2	0.3
27	53	F	Lung	BAC, M	0/9	T4N0M0	IIIB	76	dead	2.9	0.8	3.2
28	72	M	Lung	PDSCC	0/18	T2N0M0	IB	98	alive	6.0	5.0	0.5

29	63	M	Lung	MDSCC	1/25	T2N1M0	IIB	36	dead	6.4	5.5	3.0
30	63	M	Lung	MDSCC	0/22	T2N0M0	IB	97	dead	0.8	5.0	1.7
31	59	M	Lung	MDSCC	3/21	T2N1M0	IIB	97	alive	6.0	4.3	0.8
32	62	M	Lung	PDSCC	0/50	T2N0M1	IV	97	alive	6.8	5.5	3.3
33	66	M	Lung	large cell neuroendocrine carcinoma	0/38	T2N0M0	IB	12	dead	0.8	5.5	0.7
34	58	M	Lung	MDSCC	2/18	T2N1M0	IIB	95	alive	6.2	5.5	0.7
35	55	F	Lung	PDSCC	1/3	T2N1M0	IIB	79	dead	5.0	5.5	0.3
36	63	M	Lung	WDSCC	8/28	T2N2M0	IIIA	3	dead	5.7	4.3	0.3
37	81	M	Lung	WDSCC	0/17	T2N0M0	IB	94	alive	4.8	5.8	0.5
38	54	M	Lung	MDSCC	1/33	T2N1M0	IIB	25	dead	5.8	5.0	0.2
39	49	M	Lung	WDSCC	0/19	T2N0M0	IB	91	alive	6.5	5.5	0.3
40	68	M	Lung	PDSCC	0/22	T2N0M0	IB	21	dead	0.7	4.8	0.0
41	60	M	Lung	PDSCC	0/22	T2N0M0	IB	90	alive	5.3	5.2	0.0
42	65	M	Lung	large cell carcinoma	0/33	T2N0M0	IB	90	alive	5.2	4.7	0.5
43	51	M	Lung	BAC, M	1/11	T2N1M0	IIB	90	alive	5.7	5.5	0.3

44	56	M	Lung	WDSCC	4/32	T2N2M0	IIIA	25	dead	0.2	5.0	0.2
45	46	M	Lung	MDSCC	1/25	T2N1M0	IIB	5	dead	5.8	5.2	0.3
46	71	M	Lung	MDSCC	0/21	T2N0M0	IB	89	alive	5.0	4.5	0.8
47	69	M	Lung	PDSCC	0/11	T2N0M0	IB	73	dead	5.5	5.0	0.7
48	58	M	Lung	WDSCC	0/24	T2N0M0	IB	88	alive	5.7	4.3	0.7
49	62	M	Lung	BAC, NM	2/24	T1N1M0	IIA	41	dead	4.3	5.0	0.3
50	71	M	Lung	WDSCC	5/40	T2N1M0	IIB	10	dead	5.3	4.8	0.5
51	65	M	Lung	MDSCC	0/28	T3N0M0	IIB	86	alive	5.8	4.7	0.2
52	67	M	Lung	PDSCC	0/12	T2N0M0	IB	108	dead	5.7	4.5	0.7
53	64	M	Lung	PDSCC	0/14	T3N0M0	IIB	6	dead	4.7	5.0	0.8
54	63	M	Lung	carcinosarcoma	0/14	T2N0M0	IB	39	dead	4.7	5.0	0.5
55	69	M	Lung	MDSCC	0/20	T2N0M0	IB	84	alive	5.5	5.0	0.0
56	33	F	Lung	MDAC	0/6	T2N0M0	IB	83	alive	5.0	4.7	0.2
57	60	F	Lung	WDAC	22/22	T3N2M0	IIIA	14	dead	5.2	5.2	0.5
58	68	M	Lung	MDAC	0/12	T2N0M0	IB	80	alive	5.2	4.5	0.7
59	41	F	Lung	MDAC	2/15	T3N2M0	IIIA	20	dead	5.0	4.5	4.0
