# Supplementary material for: Nuclear and Cytoplasmic Accumulation of Ep-ICD Is Frequently Detected in Human Epithelial Cancers
Source: PLoS One. 2010 Nov 30;5(11):e14130. doi: 10.1371/journal.pone.0014130 (PMC2994724; doi:10.1371/journal.pone.0014130)
Supplement: Table S3 — Ep-ICD Accumulation and Clinical Parameters of Colon Cancer Patients. Abbreviations: AC: adenocarcinoma; MC: mucinous carcinoma; MD: moderately-differentiated; PD: poorly-differentiated; WD = well-differentiated. (0.08 MB PDF) [file pone.0014130.s004.pdf]

Supplementary Table S3 - Ep-ICD Accumulation and Clinical Parameters of Colon Cancer Patients

n	Age	Sex	Organ	Diagnosis	pTNM	stage	Follow-up months	Follow-up result	Cause of death	Ep-ICD Membrane	Ep-ICD Cytoplasm	Ep-ICD Nucleus
1	60	F	Ascending colon	MDAC	T3N0M0	IIA	132	alive	.	4.5	5.1	4.6
2	58	F	Rectum	MDAC	T3N1M0	IIIB	8	dead	cancer	0	0	0
3	51	M	Transverse colon	PDAC	T3N0M0	IIA	131	alive	.	2.3	4.1	4.6
4	38	F	Transverse colon	MDAC	T3N0M0	IIA	131	alive	.	2.5	5.7	5.2
5	56	M	Sigmoid colon	WDAC	T3N2M0	IIIC	27	dead	brain injury	5.8	5.8	5
6	46	F	Cecum	MDAC	T2N1M0	IIB	130	alive	.	0.83	5.5	4.7
7	61	M	Cecum	MC	T3N0M0	IIA	130	alive	.	0.83	5.67	5.67
8	40	F	Sigmoid colon	WDAC	T3N2M0	IIIC	0	lost	.	0.83	5.5	5.3
9	36	F	Descending colon	MDAC	T3N0M0	IIA	116	dead	cerebral haemorrhage	5.2	5.7	5
10	68	M	Sigmoid colon	MDAC	T3N1M0	IIIB	60	dead	cancer	4.2	5.5	4.5

11	62	F	Sigmoid colon	MDAC	T3N0M0	IIA	129	alive	.	4.7	4.3	0.3
12	65	M	Ascending colon	WDAC	T3N2M0	IIIC	129	alive	.	0.83	5.5	4.3
13	65	M	Sigmoid colon	MDAC	T3N0M0	IIA	128	alive	.	5	5.5	4.2
14	72	F	Sigmoid colon	WDAC	T3N1M0	IIIB	34	dead	cancer	0	5	0
15	63	M	Ascending colon	WDAC	T3N0M0	IIA	128	alive	.	0.3	5.5	5.7
16	73	F	Transverse colon	MDAC	T3N0M0	IIA	128	alive	.	5.5	5	4.3
17	60	M	Sigmoid colon	MDAC	T3N0M0	IIA	127	alive	.	4.3	5	5
18	65	F	Cecum	MC	T3N2M0	IIIC	127	alive	.	0.7	2.4	5.3
19	57	M	Sigmoid colon	MDAC	T3N0M1	IV	7	dead	cancer	1.8	5.5	4.8
20	58	M	Cecum	MDAC	T3N0M0	IIA	35	dead	cancer	5.5	5.5	4.8
21	56	M	Ascending colon	MC	T3N0M0	IIA	78	dead	cancer	5.7	5.2	4.7

22	57	M	Sigmoid colon	MDAC	T4N0M0	IIB	126	alive	.	0.67	4.8	5.2
23	56	M	Ascending colon	MDAC	T3N1M0	IIIB	30	dead	cancer	0.5	4.5	4.9
24	36	M	Transverse colon	MDAC	T3N2M0	IIIC	2	dead	cancer	0.3	5.5	5
25	65	F	Sigmoid colon	WDAC	T3N0M0	IIA	125	alive	.	1	4.3	4.4
26	62	M	Ascending colon	MDAC	T3N0M0	IIA	125	alive	.	0	0	0
27	57	F	Sigmoid colon	WDAC	T3N1M0	IIIB	125	alive	.	0.8	4.8	4.1
28	78	M	Sigmoid colon	WDAC	T3N1M0	IIIB	125	alive	.	0	0	0
29	45	M	Rectum	WDAC	T3N1M0	IIIB	0	dead	cancer	0.8	4.4	5.2
30	65	M	Ascending colon	WDAC	T3N1M0	IIIB	124	alive	.	0.8	2.8	5.8
31	61	M	Transverse colon	MC	T3N0M0	IIA	124	alive	.	0	2.15	5.8
32	52	M	Rectum	MDAC	T3N0M1	IV	42	dead	cancer	0.2	5.5	5.2

33	52	F	Sigmoid colon	MDAC	T2N2M0	IIIC	124	alive	.	0.5	5	5.7
34	47	F	Cecum	MDAC	T3N0M0	IIA	124	alive	.	0.7	4.2	5
35	63	F	Descending colon	MDAC	T3N1M0	IIIB	124	alive	.	0	0	0
36	53	M	Cecum	MDAC	T3N1M0	IIIB	123	alive	.	1.8	4.1	5.2
37	53	F	Transverse colon	WDAC	T3N0M0	IIA	123	alive	.	4.2	5	4.3
38	64	M	Sigmoid colon	MDAC	T3N1M0	IIIB	123	alive	.	3.9	4.4	4.2
39	59	M	Sigmoid colon	MDAC	T4N0M0	IIB	122	alive	.	4	4.5	4.2
40	57	M	Ascending colon	MDAC	T3N0M1	IV	12	dead	cancer	0	0	0
41	46	F	Ascending colon	PDAC	T3N2M0	IIIC	2	dead	unknown	0.7	0.8	4.9
42	59	M	Ascending colon	MDAC	T3N0M0	IIA	35	dead	cancer	0.8	5	4.8
43	35	F	Ascending colon	MC	T3N0M0	IIA	122	alive	.	0.83	5.2	5.2

44	42	M	Transverse colon	MDAC	T3N2M0	IIIC	122	alive	.	4.7	2.7	4.3
45	49	F	Ascending colon	MDAC	T3N2M0	IIIC	122	alive	.	4.5	4.5	4
46	76	M	Sigmoid colon	MDAC	T3N1M1	IV	57	dead	cancer	4.8	5	4.5
47	74	M	Ascending colon	MDAC	T3N0M0	IIA	122	alive	.	0.8	5	5.2
48	60	F	Rectum	MDAC	T4N0M0	IIB	122	alive	.	5	3.7	4.5
49	62	M	Sigmoid colon	MDAC	T3N2M1	IV	3	dead	cancer	0	0	0
50	62	M	Sigmoid colon	MDAC	T3N0M0	IIA	121	alive	.	0	0	0
51	57	M	Sigmoid colon	MDAC	T3N0M0	IIA	83	dead	cancer	2.8	4.7	4.8
52	56	F	Sigmoid colon	MDAC	T3N2M1	IV	11	dead	unknown	0.3	4	5.2
53	75	F	Rectum	WDAC	T3N1M0	IIIB	121	alive	.	1.3	4.7	4.8
54	58	M	Ascending colon	MDAC	T3N0M0	IIA	121	alive	.	1.8	5.5	5.3

55	56	M	Rectum	MDAC	T3N1M0	IIIB	121	alive	.	0.8	4.2	4.5
56	35	M	Ascending colon	MDAC	T2N1M0	IIIA	120	alive	.	0.5	5	4.3
57	34	M	Transverse colon	MDAC	T3N1M0	IIIB	120	alive	.	0.2	4.5	4.5
58	60	M	Descending colon	WDAC	T3N0M0	IIA	120	alive	.	0.7	5	4.7
