# Supplementary material for: Nuclear and Cytoplasmic Accumulation of Ep-ICD Is Frequently Detected in Human Epithelial Cancers
Source: PLoS One. 2010 Nov 30;5(11):e14130. doi: 10.1371/journal.pone.0014130 (PMC2994724; doi:10.1371/journal.pone.0014130)
Supplement: Table S4 — Ep-ICD Accumulation and Clinical Parameters of Breast Cancer Patients. Abbreviations: IDC: infiltrating duct carcinoma. (0.17 MB PDF) [file pone.0014130.s005.pdf]

Supplementary Table S4 – Ep-ICD Accumulation and Clinical Parameters of Breast Cancer Patients

n	Age	Sex	Organ	Diagnosis	pTNM	Stage	LN*	ER*	PR*	P53	Histologic grade**	Tumor Size	Follow-up months	Follow-up result	Ep-ICD Nucleus	Ep-ICD Cytoplasm	Ep-ICD Membrane
1	28	F	Breast	IDC	T3N3aM1	IV	12/15	—	+	—	II	6.0 cm	47	Dead	5.2	4.7	0.7
2	28	F	Breast	IDC	T3N3aM1	IV	12/15	—	+	—	II	6.0 cm	47	dead	5	4.8	0.3
3	47	F	Breast	IDC	T2N2aM0	IIIA	5/22	—	—	+	III	3.5 cm	107	alive	4.8	5.3	0.7
4	47	F	Breast	IDC	T2N2aM0	IIIA	5/22	—	—	+	III	3.5 cm	107	alive	5.2	5.2	1.2
5	39	F	Breast	IDC	T2N0M0	IIA	0/16	—	+	+	II	3.5 cm	107	alive	5.2	5.2	0.7
6	39	F	Breast	IDC	T2N0M0	IIA	0/16	—	+	+	II	3.5 cm	107	alive	5.2	5	1
7	48	F	Breast	IDC	T2N2aM0	IIA	5/22	—	—	—	III	3.5 cm	105	alive	4.7	4.7	0.7
8	48	F	Breast	IDC	T2N2aM0	IIA	5/22	—	—	—	III	3.5 cm	105	alive	5	4.8	0.3
9	60	F	Breast	IDC	T2N0M0	IIA	0/9	—	—	+	II	2.7 cm	104	alive	4.9	4.9	0.7
10	60	F	Breast	IDC	T2N0M0	IIA	0/9	—	—	+	II	2.7 cm	104	alive	4.8	4	0.3
11	54	F	Breast	IDC	T3N1aM1	IV	01/16	+	—	—	II	6.5 cm	103	alive	4.7	5	1.3
12	54	F	Breast	IDC	T3N1aM1	IV	01/16	+	—	—	II	6.5 cm	103	alive	4.7	5	1.2
13	43	F	Breast	IDC	T3N0M0	IIB	0/9	—	—	+	I	5.0 cm	103	alive	0	0	0
14	43	F	Breast	IDC	T3N0M0	IIB	0/9	—	—	+	I	5.0 cm	103	alive	0	0	0
15	42	F	Breast	IDC	T2N2aM0	IIIA	5/19	+	+	—	II	3.0 cm	103	alive	5.7	5	0.2
16	42	F	Breast	IDC	T2N2aM0	IIIA	5/19	+	+	—	II	3.0 cm	103	alive	6.2	5.2	0.3
17	40	F	Breast	IDC	T2N3aM0	IIIC	18/21	+	+	—	II	2.5 cm	58	dead	5.2	5.2	0.2
18	40	F	Breast	IDC	T2N3aM0	IIIC	18/21	+	+	—	II	2.5 cm	58	dead	4.6	4.4	0.8
19	41	F	Breast	IDC	T2N1aM0	IIB	2/18	—	+	+	I	4.0 cm	102	alive	5.3	5.3	0.3
20	41	F	Breast	IDC	T2N1aM0	IIB	2/18	—	+	+	I	4.0 cm	102	alive	5.7	6	1.3
21	57	F	Breast	IDC	T2N3aM0	IIIC	15/24	—	—	+	II	2.5 cm	11	dead	0	0	0

22	57	F	Breast	IDC	T2N3aM0	IIIC	15/24	—	—	+	II	2.5 cm	11	dead	5.7	5.8	0.2
23	44	F	Breast	IDC	T2N3aM0	IIIC	28/28	—	—	+	III	3.5 cm	101	alive	5	`	0.7
24	44	F	Breast	IDC	T2N3aM0	IIIC	28/28	—	—	+	III	3.5 cm	101	alive	5	5	0.7
25	84	F	Breast	IDC	T2N3aM0	IIA	0/18	+	+	—	I	2.5 cm	101	alive	5.7	5.7	0.8
26	84	F	Breast	IDC	T2N3aM0	IIA	0/18	+	+	—	I	2.5 cm	101	alive	5.8	5.8	1.3
27	52	F	Breast	IDC	T2N1aM0	IIB	1/14	+	+	—	II	II	97	alive	5	5	0.8
28	52	F	Breast	IDC	T2N1aM0	IIB	1/14	+	+	—	II	II	97	alive	5.5	5	0.5
29	40	F	Breast	IDC	T2N0M0	IIA	0/10	—	—	—	III	III	39	dead	5.2	4.8	0.3
30	40	F	Breast	IDC	T2N0M0	IIA	0/10	—	—	—	III	III	39	dead	0	0	0
31	66	F	Breast	IDC	T2N0M0	IIA	.	.	.	.	.	.	.	.	0.2	4.8	1.8
32	43	F	Breast	IDC	T3N2aM0	IIIA	.	.	.	.	.	.	.	.	0.2	5	2.3
33	58	F	Breast	IDC	T2N3aM0	IIIC	.	.	.	.	.	.	.	.	5.5	2.5	2
34	46	F	Breast	IDC	T3N1aM0	IIIA	.	.	.	.	.	.	.	.	2.2	3.7	0.2
35	71	F	Breast	IDC	T3N1aM0	IIIA	.	.	.	.	.	.	.	.	5.2	5	0.5
36	37	F	Breast	IDC	T3N3aM0	IIIC	.	.	.	.	.	.	.	.	ND	ND	ND
37	53	F	Breast	IDC	T3N2aM0	IIIA	.	.	.	.	.	.	.	.	ND	ND	ND
38	30	F	Breast	IDC	T3N0M0	IIB	.	.	.	.	.	.	.	.	5.3	4.7	0.7
39	47	F	Breast	IDC	T2N1aM0	IIB	.	.	.	.	.	.	.	.	5.3	5	0.5
40	44	F	Breast	IDC	T2N0M0	IIA	.	.	.	.	.	.	.	.	5.3	5	1.5
41	28	F	Breast	IDC	.	.	.	.	.	.	.	.	.	.	1.5	1.4	0
42	28	F	Breast	IDC	.	.	.	.	.	.	.	.	.	.	0	0.9	0

43	47	F	Breast	IDC	.	.	.	.	.	.	.	.	.	.	0	0	0
44	47	F	Breast	IDC	.	.	.	.	.	.	.	.	.	.	1	1	0
45	39	F	Breast	IDC	.	.	.	.	.	.	.	.	.	.	0	1	0
46	39	F	Breast	IDC	.	.	.	.	.	.	.	.	.	.	1	0.8	0
47	48	F	Breast	IDC	.	.	.	.	.	.	.	.	.	.	0.2	0.4	0
48	48	F	Breast	IDC	.	.	.	.	.	.	.	.	.	.	0.9	1.4	0
49	60	F	Breast	IDC	.	.	.	.	.	.	.	.	.	.	0.9	1.2	0
50	60	F	Breast	IDC	.	.	.	.	.	.	.	.	.	.	1.4	1.5	0
51	54	F	Breast	IDC	.	.	.	.	.	.	.	.	.	.	0.8	1.2	0
52	54	F	Breast	IDC	.	.	.	.	.	.	.	.	.	.	0	0.2	0
53	43	F	Breast	IDC	.	.	.	.	.	.	.	.	.	.	0.2	0.2	0
54	43	F	Breast	IDC	.	.	.	.	.	.	.	.	.	.	1.7	1.9	0
55	42	F	Breast	IDC	.	.	.	.	.	.	.	.	.	.	0.4	0.4	0
56	42	F	Breast	IDC	.	.	.	.	.	.	.	.	.	.	0	0	0
57	40	F	Breast	IDC	.	.	.	.	.	.	.	.	.	.	0	0	0
58	40	F	Breast	IDC	.	.	.	.	.	.	.	.	.	.	0.2	0.2	0
59	41	F	Breast	IDC	.	.	.	.	.	.	.	.	.	.	1	1.2	0
60	41	F	Breast	IDC	.	.	.	.	.	.	.	.	.	.	1.4	1.5	0

61	57	F	Breast	IDC	.	.	.	.	.	.	.	.	.	.	0.9	0.9	0
62	57	F	Breast	IDC	.	.	.	.	.	.	.	.	.	.	0.2	0.4	0
63	44	F	Breast	IDC	.	.	.	.	.	.	.	.	.	.	0	0.3	0
64	44	F	Breast	IDC	.	.	.	.	.	.	.	.	.	.	0	0	0
65	84	F	Breast	IDC	.	.	.	.	.	.	.	.	.	.	0	0	0
66	84	F	Breast	IDC	.	.	.	.	.	.	.	.	.	.	.	.	.
67	52	F	Breast	IDC	.	.	.	.	.	.	.	.	.	.	.	.	.
68	52	F	Breast	IDC	.	.	.	.	.	.	.	.	.	.	.	.	.
69	40	F	Breast	IDC	.	.	.	.	.	.	.	.	.	.	.	.	.
70	40	F	Breast	IDC	.	.	.	.	.	.	.	.	.	.	.	.	.
