# Supplementary material for: Nuclear and Cytoplasmic Accumulation of Ep-ICD Is Frequently Detected in Human Epithelial Cancers
Source: PLoS One. 2010 Nov 30;5(11):e14130. doi: 10.1371/journal.pone.0014130 (PMC2994724; doi:10.1371/journal.pone.0014130)
Supplement: Table S5 — Ep-ICD Accumulation and Clinical Parameters of Liver Cancer Patients. (0.02 MB PDF) [file pone.0014130.s006.pdf]

Supplementary Table S5 - Ep-ICD Accumulation and Clinical Parameters of Liver Cancer Patients

n	Organ	Diagnosis	Age	Sex	pTNM	Stage	Ep-ICD Nucleus	Ep-ICD Cytoplasm	Ep-ICD Membrane
1	Liver	combined hepatocellular and cholangiocarcinoma	32	M	T3N0M0	IIIA	6.0	5.5	0.2
2	Liver	hepatocellular carcinoma	57	M	T3N0M0	IIIA	5.3	4.8	1.0
3	Liver	hepatocellular carcinoma	58	F	T3N0M0	IIIA	4.5	1.9	4.5
4	Liver	hepatocellular carcinoma	20	M	T4N0M0	IIIB	5.3	5.0	0.7
5	Liver	hepatocellular carcinoma	66	F	T1N0M0	I	5.0	4.8	0.5
6	Liver	hepatocellular carcinoma	61	M	T1N0M0	I	5.0	4.8	0.7
7	Liver	hepatocellular carcinoma	52	M	T3N0M0	IIIA	4.5	4.8	0.0
8	Liver	combined hepatocellular and cholangiocarcinoma	50	M	T3N1M0	IIIC	4.5	4.8	0.0
9	Liver	hepatocellular carcinoma	56	M	T3N0M0	IIIA	4.7	5.0	0.3
