# Supplementary material for: Nuclear and Cytoplasmic Accumulation of Ep-ICD Is Frequently Detected in Human Epithelial Cancers
Source: PLoS One. 2010 Nov 30;5(11):e14130. doi: 10.1371/journal.pone.0014130 (PMC2994724; doi:10.1371/journal.pone.0014130)
Supplement: Table S6 — Ep-ICD Accumulation and Clinical Parameters of Bladder Cancer Patients. Abbreviations: TCC: transitional cell carcinoma. (0.02 MB PDF) [file pone.0014130.s007.pdf]

Supplementary Table S6 - Ep-ICD Accumulation and Clinical Parameters of Bladder Cancer Patients

n	Organ	Diagnosis	Age	Sex	pTNM	Stage	Ep-ICD Nucleus	Ep-ICD Cytoplasm	Ep-ICD Membrane
1	Bladder	mucinous adenocarcinoma from urachal remnant	53	M	T4bN0M0	IV	2.4	2.4	.5
2	Bladder	TCC	60	M	T3bN1M0	IV	4.3	4.7	.2
3	Bladder	TCC	60	M	T3bN1M0	IV	4.7	4.8	.3
4	Bladder	TCC	65	M	T4aN1M1	IV	4.7	4.5	.0
5	Bladder	TCC	74	M	T1N0M0	I	5.0	5.0	1.0
6	Bladder	TCC	63	F	T1N0M0	I	4.7	5.0	1.0
7	Bladder	TCC	58	M	T3NxM0	III	4.2	5.0	.8
8	Bladder	TCC	46	M	T1N0M0	I	6.0	4.8	.3
9	Bladder	TCC	76	M	T3NxM0	III	5.8	4.8	.3
10	Bladder	TCC	63	M	T3bN0M0	III	5.0	4.3	.3
