# Supplementary material for: Nuclear and Cytoplasmic Accumulation of Ep-ICD Is Frequently Detected in Human Epithelial Cancers
Source: PLoS One. 2010 Nov 30;5(11):e14130. doi: 10.1371/journal.pone.0014130 (PMC2994724; doi:10.1371/journal.pone.0014130)
Supplement: Table S7 — Ep-ICD Accumulation and Clinical Parameters of Ovarian Cancer Patients. Abbreviations: MAC: mucinous adenocarcinoma; MD: moderately differentiated; PD: poorly differentiated; SAC: serous adenocarcinoma. (0.02 MB PDF) [file pone.0014130.s008.pdf]

Supplementary Table S7 - Ep-ICD Accumulation and Clinical Parameters of Ovarian Cancer Patients

n	Organ	Diagnosis	Age	Sex	pTNM	Stage	Ep-ICD Nucleus	Ep-ICD Cytoplasm	Ep-ICD Membrane
1	Ovary	SAC	57	F	T3bN1M0	IIIC	4.8	5.5	0
2	Ovary	SAC, MD	48	F	T3cN0M0	IIIC	4.2	5.0	1
3	Ovary	Serous Surface Papillary Carcinoma, PD	60	F	T2cN0M0	IIC	4.8	5.2	1
4	Ovary	SAC, MD	41	F	T2cN1M0	IIIC	5.0	5.0	1
5	Ovary	SAC	64	F	T3cN0M0	IIIC	4.2	5.0	0
6	Ovary	SAC	60	F	T3cN1M0	IIIC	4.5	5.0	0
7	Ovary	SAC	54	F	T3aN0M0	IIIA	4.7	5.5	0
8	Ovary	SAC	58	F	T2cN0M0	IIC	4.8	5.0	1
9	Ovary	Mucinous Adenocarcinoma	16	F	T1aN0M0	IA	5.7	4.7	1
10	Ovary	SAC, PD	57	F	T3cN0M0	IIIC	4.2	4.7	4
