# Supplementary material for: Nuclear and Cytoplasmic Accumulation of Ep-ICD Is Frequently Detected in Human Epithelial Cancers
Source: PLoS One. 2010 Nov 30;5(11):e14130. doi: 10.1371/journal.pone.0014130 (PMC2994724; doi:10.1371/journal.pone.0014130)
Supplement: Table S8 — Ep-ICD Accumulation and Clinical Parameters of Pancreatic Cancer Patients. Abbreviations: MDDAC: moderately differentiated ductal adenocarcinoma; PDDAC: poorly differentiated ductal adenocarcinoma. (0.02 MB PDF) [file pone.0014130.s009.pdf]

Supplementary Table S8 - Ep-ICD Accumulation and Clinical Parameters of Pancreatic Cancer Patients

n	Organ	Diagnosis	Age	Sex	pTNM	Stage	Ep-ICD Nucleus	Ep-ICD Cytoplasm	Ep-ICD Membrane
1	Pancreas	MDDAC	66	M	T4N1M0	III	2.7	2.7	0.7
2	Pancreas	MDDAC	71	M	T3N1M0	IIB	3.8	3.8	0.7
3	Pancreas	MDDAC	68	M	T3N1M0	IIB	4.0	3.5	0.3
4	Pancreas	MDDAC	45	M	T3N1M0	IIB	3.5	4.0	0.0
5	Pancreas	MDDAC	64	F	T4N0M0	III	5.2	5.0	0.8
6	Pancreas	MDDAC	64	F	T3N1M0	IIB	2.5	2.5	1.0
7	Pancreas	PDDAC	69	M	T4NxM0	III	1.8	2.5	0.8
8	Pancreas	MDDAC	46	M	T3N1M0	IIB	4.2	0.8	0.8
9	Pancreas	MDDAC	54	M	T3N1M0	IIB	3.7	0.7	0.8
10	Pancreas	MDDAC	40	M	T3N1M0	IIB	3.5	4.0	1.0
