# Supplementary material for: Nuclear and Cytoplasmic Accumulation of Ep-ICD Is Frequently Detected in Human Epithelial Cancers
Source: PLoS One. 2010 Nov 30;5(11):e14130. doi: 10.1371/journal.pone.0014130 (PMC2994724; doi:10.1371/journal.pone.0014130)
Supplement: Table S9 — Ep-ICD Accumulation and Clinical Parameters of HNSCC Patients. Abbreviations: MD: moderately differentiated; PD: poorly differentiated; SCC: squamous cell carcinoma; WD: well differentiated. (0.08 MB PDF) [file pone.0014130.s010.pdf]

Supplementary Table S9 - Ep-ICD Accumulation and Clinical Parameters of HNSCC Patients

N	Gender	Tissue Type	pTNM	Diagnosis	Follow-Up Result	Follow-Up Time (Months)	Ep-ICD Nucleus	Ep-ICD Cytoplasm	Ep-ICD Membrane
1	M	Head and Neck	T4N1M0	WDSCC	DEAD	9	0	0	0
2	F	Head and Neck	T4N1M0	WDSCC	RECURRENCE	14	0	0	0
3	F	Head and Neck	T3N2bM0	WDSCC	DEAD	13	5	6	0
4	M	Head and Neck	T4N2bM0	MDSCC	DEAD	8	7	7	0
5	M	Head and Neck	T4N0M0	WDSCC	NAD	31	6	5	0
6	F	Head and Neck	T3N0M0	WDSCC	NAD	30	4	5	0
7	M	Head and Neck	T3N2aM0	MDSCC	NAD	28	0	3	0
8	M	Head and Neck	T3N2aM0	WDSCC	RECURRENCE	7	6	7	0
9	M	Head and Neck	T3N2aM0	WDSCC	NAD	29	0	0	0
10	M	Head and Neck	T4N1M0	MDSCC	DEAD	8	5	4	0
11	M	Head and Neck	T3N0M0	MDSCC	NAD	28	7	7	0
12	M	Head and Neck	T4N1M0	WDSCC	NAD	28	7	7	0
13	M	Head and Neck	T4N1M0	WDSCC	NAD	18	7	7	0

14	M	Head and Neck	T4N2cM0	MDSCC	DEAD	6	0	5	0
15	M	Head and Neck	T2N2aM0	WDSCC	RECURRENCE	6	5	5	0
16	M	Head and Neck	T4N2bM0	MDSCC	RECURRENCE	6	0	0	0
17	M	Head and Neck	T3N1M0	WDSCC	NAD	24	5	6	0
18	M	Head and Neck	T4N0M0	WDSCC	RECURRENCE	8	2	6	0
19	F	Head and Neck	T3N0M0	WDSCC	NAD	23	5	4	0
20	M	Head and Neck	T4N1M0	MDSCC	DEAD	3	7	7	0
21	M	Head and Neck	T4N1M0	WDSCC	NAD	21	0	5	0
22	M	Head and Neck	T4N2aM0	MDSCC	DEAD	3	4	6	0
23	M	Head and Neck	T3N2bM0	WDSCC	DEAD	3	5	5	0
24	M	Head and Neck	T2N2bM0	MDSCC	DEAD	3	6	6	0
25	F	Head and Neck	T3N1M0	WDSCC	DEAD	18	7	6	0
26	M	Head and Neck	T3N0M0	MDSCC	NAD	19	7	5	0
27	M	Head and Neck	T4N0M0	WDSCC	RECURRENCE	11	5	6	0
28	F	Head and Neck	T3N2M0	MDSCC	NAD	17	7	5	0
29	F	Head and Neck	T2N1Mo	MDSCC	NAD	17	5	5	0

30	M	Head and Neck	T3N2bM0	MDSCC	NAD	16	5	6	0
31	M	Head and Neck	T3N2bM0	WDSCC	DEAD	8	3	3	0
32	M	Head and Neck	T2N1M0	WDSCC	NAD	16	7	6	0
33	M	Head and Neck	T4N2cM0	MDSCC	NAD	16	7	7	0
34	M	Head and Neck	T2N1M0	WDSCC	NAD	17	7	7	0
35	M	Head and Neck	T1N1M0	WDSCC	NAD	15	7	7	0
36	M	Head and Neck	T4N0M0	WDSCC	NAD	14	7	6	0
37	F	Head and Neck	T3N0M0	WDSCC	NAD	14	4	6	0
38	M	Head and Neck	T4N2cM0	MDSCC	DEAD	9	6	6	0
39	M	Head and Neck	T3N0M0	WDSCC	NAD	11	6	7	0
40	M	Head and Neck	T3N0M0	MDSCC	NAD	11	0	0	0
41	M	Head and Neck	T3N0M0	WDSCC	RECURRENCE	6	5	5	0
42	F	Head and Neck	T4N1M0	WDSCC	DEAD	2	0	5	0
43	M	Head and Neck	T4N1M0	MDSCC	NAD	12	0	0	0
44	M	Head and Neck	T3N2bM0	WDSCC	NAD	11	6	6	0
45	M	Head and Neck	T4N2cM0	WDSCC	NAD	12	4	5	0

46	M	Head and Neck	T4N1M0	MDSCC	DEAD	7	6	6	0
47	M	Head and Neck	T4N0M0	MDSCC	NAD	10	6	5	0
48	M	Head and Neck	T4N0M0	WDSCC	NAD	10	0	0	0
49	M	Head and Neck	T3N2bM0	WDSCC	DEAD	9	5	5	0
50	M	Head and Neck	T4N2M0	MDSCC	DEAD	12	0	0	0
51	M	Head and Neck	T2N2M0	MDSCC	RECURRENCE	20	0	0	0
52	M	Head and Neck	T3N1M0	MDSCC	RECURRENCE	3	0	0	0
53	M	Head and Neck	T4N0M0	MDSCC	DEAD	14	7	7	0
54	F	Head and Neck	T3N1M0	WDSCC	NAD	27	0	0	0
55	M	Head and Neck	T4N1M0	WDSCC	DEAD	7	0	0	0
56	F	Head and Neck	T4N2M0	MDSCC	DEAD	6	0	4	0
57	M	Head and Neck	T2N1M0	WDSCC	DEAD	8	0	0	0
58		Head and Neck		Normal			0	0	0
59		Head and Neck		Normal			0	0	0
60		Head and Neck		Normal			0	0	0
61		Head and Neck		Normal			4	4	0

62		Head and Neck		Normal			0	0	0
63		Head and Neck		Normal			0	0	0
64		Head and Neck		Normal			2	3	0
65		Head and Neck		Normal			0	0	0
66		Head and Neck		Normal			0	0	0
67		Head and Neck		Normal			0	0	0
68		Head and Neck		Normal			0	3	0
69		Head and Neck		Normal			0	0	0
70		Head and Neck		Normal			0	0	0
71		Head and Neck		Normal			0	0	0
72		Head and Neck		Normal			0	0	0
73		Head and Neck		Normal			0	0	0
74		Head and Neck		Normal			0	0	0
75		Head and Neck		Normal			0	0	0
76		Head and Neck		Normal			0	0	0
77		Head and Neck		Normal			0	0	0
