# Supplementary material for: Nuclear and Cytoplasmic Accumulation of Ep-ICD Is Frequently Detected in Human Epithelial Cancers
Source: PLoS One. 2010 Nov 30;5(11):e14130. doi: 10.1371/journal.pone.0014130 (PMC2994724; doi:10.1371/journal.pone.0014130)
Supplement: Table S10 — Ep-ICD Accumulation and Clinical Parameters of ESCC Patients. Abbreviations: AC: adenocarcinoma; MD: moderately differentiated; PD: poorly differentiated; SCC: squamous cell carcinoma; WD: well differentiated. (0.10 MB PDF) [file pone.0014130.s011.pdf]

Supplementary Table S10 - Ep-ICD Accumulation and Clinical Parameters of ESCC Patients

n	Tissue Type	Histopath	TNM	Ep-ICD Nucleus	Ep-ICD Cytoplasm	Ep-ICD Membrane
1	Esophagus	PDSCC	T3N1M0	0	6	0
2	Esophagus	WDSCC	T2N0M0	0	5	0
3	Esophagus	WDSCC	T3N1M0	5	6	0
4	Esophagus	WDSCC	T2N0M0	0	4	0
5	Esophagus	WDSCC	T3N1M0	0	3	0
6	Esophagus	WDSCC	T2N1M0	0	6	0
7	Esophagus	WDSCC	T2N1M0	0	0	0
8	Esophagus	WDSCC	–	0	7	0
9	Esophagus	WDSCC	T2N1M0	0	0	0
10	Esophagus	SCC	T2N0M0	0	5	0
11	Esophagus	SCC	T2N0M0	0	7	0
12	Esophagus	SCC	–	7	7	0
13	Esophagus	SCC	T2N0M0	0	4	0
14	Esophagus	SCC	T4N1M0	7	7	0
15	Esophagus	SCC	T3N1M0	3	0	0
16	Esophagus	SCC	–	0	0	0
17	Esophagus	SCC	–	6	6	0
18	Esophagus	SCC	T3N1M0	5	6	0
19	Esophagus	SCC	T2N0M0	0	0	0
20	Esophagus	PDSCC	T3N1M0	0	0	0
21	Esophagus	PDSCC	T3N1M0	4	5	0
22	Esophagus	PDSCC	T3N1M0	0	0	0
23	Esophagus	PDSCC	T4N1M0	6	7	0
24	Esophagus	PDSCC	T4N1M0	7	7	0
25	Esophagus	PDSCC	T3N1M0	0	7	0
26	Esophagus	PDSCC	T3N1M0	0	7	0
27	Esophagus	PDSCC	T3N1M0	0	0	0
28	Esophagus	PDSCC	T3N1M0	0	0	0
29	Esophagus	MDSCC	T2N0M0	0	0	0
30	Esophagus	MDSCC	T3N1M0	0	5	0
31	Esophagus	MDSCC	T3N0M0	0	6	0
32	Esophagus	MDSCC	T3N1M0	0	**0**	0
33	Esophagus	MDSCC	T3N0M0	0	0	0
34	Esophagus	MDSCC	T2N1M0	5	6	0
35	Esophagus	MDSCC	T3N1M0	5	7	0
36	Esophagus	MDSCC	T3N1M0	7	7	0
37	Esophagus	MDSCC	T3N1M0	5	6	0
38	Esophagus	MDSCC	T3N1M0	0	7	0
39	Esophagus	Dys	–	0	0	0
40	Esophagus	AC	T2N0M0	0	5	0
41	Esophagus	AC	T3N1Mx	6	6	0
42	Esophagus	AC	T3N0M0	4	6	0

43	Esophagus	AC	T3N1M0	6	7	0
44	Esophagus	AC	T4N1M0	7	7	0
45	Esophagus	AC	—	4	6	0
46	Esophagus	AC	T4N1M0	0	7	0
47	Esophagus	Normal	—	0	0	0
48	Esophagus	Normal	—	0	0	0
49	Esophagus	Normal	—	3	5	0
50	Esophagus	Normal	—	0	4	0
51	Esophagus	Normal	—	0	3	0
52	Esophagus	Normal	—	0	0	0
53	Esophagus	Normal	—	0	0	0
54	Esophagus	Normal	—	0	0	0
55	Esophagus	Normal	—	0	0	0
56	Esophagus	Normal	—	0	0	0
57	Esophagus	Normal	—	0	3	0
58	Esophagus	Normal	—	0	4	0
59	Esophagus	Normal	—	0	**0**	0
60	Esophagus	Normal	—	0	0	0
61	Esophagus	Normal	—	7	7	0
62	Esophagus	Normal	—	4	4	0
63	Esophagus	Normal	—	0	4	0
64	Esophagus	Normal	—	0	0	0
65	Esophagus	Normal	—	0	0	0
66	Esophagus	Normal	—	0	0	0
